# Behavioral choice of manufacturers, recyclers and customers in Trade-In Programs

**DOI:** 10.1371/journal.pone.0316344

**Published:** 2024-12-30

**Authors:** Chan He, Qianru An

**Affiliations:** Business School, Shanghai Dianji University, Shanghai, Pudong, China; Brno University of Technology Faculty of Civil Engineering: Vysoke uceni technicke v Brne Fakulta stavebni, CZECHIA

## Abstract

This study investigates the behavioral choices of manufacturers, recyclers, and customers in Trade-In Programs designed to promote recycling and environmental sustainability. Using a manufacturer-led evolutionary game model, the research explores how factors such as government policies, market demand, financial incentives, and the simplicity of participation impact stakeholder engagement in these programs. Numerical simulations were conducted to analyze the effects of specific parameters on each participants’ willingness to participate. Results show that increased subsidies and investment in innovation by manufacturers significantly enhance recycler and customer participation in ethic Trade-In Programs. Conversely, higher revenue-sharing by manufacturers tends to reduce engagement from both parties, while manufacturers receiving substantial brand and social benefits alongside government subsidies to encourage broader stakeholder involvement. These findings offer critical insights for designing effective Trade-In Programs, supporting sustainable recycling practices and circular economy principles. The study provides actionable guidance for policymakers and industry leaders aiming to boost participation in environmentally focused recycling initiatives, laying a foundation for more sustainable industrial practices.

## Introduction

In recent years, rapid technological advancements and economic expansion have accelerated product obsolescence, resulting in a growing accumulation of outdated consumer goods [1, 2]. To address the environmental challenges posed by electronic waste, China has introduced policies that encourage Trade-In Programs (TIPs) for the recycling of end-of-life products [3]. Typically supported by government and manufacturer-provided subsidies or incentives, TIPs enable consumers to exchange old items for new, energy-efficient products [1–7].

Manufacturers in China, such as Haier and Apple, have responded positively to TIPs by offering discounts and subsidies to incentivize consumers to trade in their old products [4]. These initiatives not only promote product updates but also align with Extended Producer Responsibility (EPR) policies, which hold manufacturers accountable for the environmental impact of their products [1, 5]. TIPs allow manufacturers to build environmentally friendly supply chains, strengthen their brand reputation, and reduce marketing costs by leveraging government support [2–5]. However, the success of TIPs depends on the engagement of all stakeholders—manufacturers, recyclers, and consumers—each with distinct motivations and constraints that influence their participation [2, 6–13].

Recyclers also benefit from TIPs, gaining more orders and improving recycling efficiency through partnerships with manufacturers and retailers [14–16]. For example, companies like Hi Recycling have successfully collaborated with retail giants and mid-sized manufacturers, such as Midea and Daikin, to enhance resource utilization effectiveness and promote sustainable consumer behaviors through joint initiatives. Evidence suggests that TIPs effectively increase collection rates for obsolete products, influencing production and sales processes of recycled materials [16–20]. These programs not only encourage consumers to upgrade their appliances but also enhance the quality of life by keeping them informed about market trends and product advancements [20–22]. As a result, TIPs stimulate consumer demand and support structural reforms in supply chains [23–29].

According to Li [11], the success of TIPs hinges on well-designed Trade-In policies and accurate return-flow predictions to support operational decision-making. Despite TIPs’ potential to foster a sustainable recycling ecosystem, understanding the behaviors and decision-making processes of recyclers and consumers within these programs remains challenging [1, 2, 5, 6, 17, 22]. To address this gap, this study employs an evolutionary game model (EGM) to examine the strategic interactions between manufacturers, recyclers, and customers within TIPs. EGM provides a dynamic framework for examining how stakeholders adapt their strategies over time based on incentives, costs, and potential gains, enabling identification of the most effective factors—such as subsidies, revenue-sharing, and innovation investments—for promoting active TIP participation. Specifically, this study addresses three research questions: (1) What factors influence the strategic decision-making of manufacturers, recyclers, and consumers? (2) How do these entities adapt their strategies over time, and which strategies are most effective? (3) How do variables such as costs, innovation investment, subsidies, and revenue-sharing ratios affect manufacturers’ incentive-related decisions?

This approach facilitates a comprehensive analysis of behavioral strategies as influenced by factors such as asymmetric information, intent strategies, and decision-making rights. The model considers evolutionary conditions of each participant, examining their survival and adaptation within the TIP system under different strategic scenarios. Through numerical simulations, we evaluate the impact of key parameters, including government subsidies, innovation investments, and revenue-sharing ratios, on stakeholder behavior. Our findings indicate that increased subsidies and investment in innovation by manufacturers lead to greater participation from recyclers and customers. Conversely, higher revenue-sharing ratios reduce engagement. These insights provide actionable recommendations for enhancing stakeholder participation in TIPs, contributing to sustainable recycling practices and the circular economy. Ultimately, this research underscores the importance of well-designed TIPs in advancing sustainability in the recycling sector and offers valuable guidance for policymakers and industry leaders.

## Theoretical development

### Trade-In Programs

#### The role of Trade-In Programs in sustainable recycling

Trade-In Programs (TIPs) are increasingly utilized to promote sustainable practices and circular economy objectives by incentivizing customers to return used products for discounts on new purchases [1–11]. TIPs enable manufacturers to reclaim valuable materials, reduce waste, and contribute to environmental sustainability by facilitating the remanufacturing of goods. Additionally, TIPs strengthen customer loyalty and support a dual agenda of enhancing sustainable practices and competitive market advantage [1, 9–11]. Van [17] proposed that Trade-In services can be utilized by businesses to increase the frequency of consumer purchases. Similarly, Park [18] pointed out that TIPs create greater enthusiasm among consumers to buy new products. Choi [19] emphasized that incorporating extended consumer responsibility through TIPs expands market reach and increases profitability, suggesting TIPs as effective mechanisms for sustainable growth. Yin [20] found that appropriate pricing strategies within TIPs can enhance both profit margins and customer purchase frequency. Numerous studies have highlighted the multi-faceted benefits of TIPs, including the promotion of technological advancements, accelerating product iteration, enhancing consumer satisfaction, and improving corporate competitiveness [2, 6–12]. Although these studies illustrate the effectiveness of TIPs in promoting recycling and increasing consumption, the long-term sustainability impacts of TIPs, particularly in terms of empirical evidence linking specific TIP structures and incentives directly to stakeholder engagement to the circular economy, are less explored.

#### Stakeholder dynamics in Trade-In Programs

Studies exploring the dynamics of TIPs suggest that these programs can foster technological innovation and product iteration, thus increasing sales and customer engagement. Liu [9], Xiao [10], and Li [11] found that TIPs are especially effective when there is a significant value difference between older and newer products, as this incentivizes customers to upgrade for improved technology. Levinthal [12] and Tang [13] also found that TIP services increase customer willingness to adopt new technology, highlighting TIPs as a strategic tool for advancing product evolution. Li [14] and Han [15] explored optimal conditions for implementing TIPs, identifying product robustness as a key factor in motivating consumer participation. Dou [16] showed that higher levels of manufacturer investment in technological innovation attract more customers to TIPs, reinforcing that customer participation is highly responsive to product innovation. While these studies examine TIPs’ effects on consumer behavior, there remains limited research on the combined impact of TIP incentives on multiple stakeholders (e.g., manufacturers, recyclers, and customers) in a unified system. This study fills this gap by analyzing the dynamics between these groups and exploring the influence of manufacturers’ technological investments on stakeholder participation.

#### Manufacturer-led Trade-In mechanisms and strategic considerations

Research into TIPs has extensively covered pricing tactics and execution approaches. Zhao [21] explored the mechanisms through which manufacturers effectively implement TIPs and improve their outcomes. Xiao [1] explored optimal TIP timing, recommending early-stage TIP implementation to maximize profitability. Li [22], considered the impact of limited core availability of remanufacturing due to the collection of old products on TIP. Although these studies provide insights into TIP implementation from a manufacturer’s perspective, there is limited research on how manufacturers’ decisions impact the behavior of recyclers and customers within an integrated system [29]. This study addresses this gap by incorporating manufacturers, recyclers, and customers into a unified model, allowing for an examination of interdependent decision-making in TIP settings. The incentive mechanism is designed with a focus on manufacturers’ input in technological innovation and their provision of subsidies to recyclers and customers.

### The application of EGM

EGM is particularly relevant to analyzing strategic behavior in TIPs, as it accommodates bounded rationality and dynamic adjustments among participants. Unlike traditional game theory, which assumes complete rationality, EGM recognizes cognitive limitations, making it well-suited for studying the adaptive strategies in multi-stakeholder environments such as TIPs [23, 24, 26, 29]. EGM applications in sustainability research reveal that governments often drive green innovation through regulation and subsidies. For example, Li [25] and Wang [26] demonstrated that government-led policies incentivize green technology adoption, which can be analyzed using EGM models. Zhao [27] and Yuan [28] applied EGM to study interactions between the government, enterprises, and other stakeholders, finding that EGM effectively captures the dynamic nature of policy-driven innovation. Meanwhile, Guo [29] employed an EGM to investigate how the government can promote technological innovation in the green development of manufacturers.

In this study, EGM is applied to model the interactions between manufacturers, recyclers, and customers within a TIP framework. EGT is well-suited for this analysis because it models how stakeholders adapt their strategies over time, responding to incentives and penalties that either promote or inhibit cooperation. In these programs, manufacturers, recyclers, and consumers operate under varying incentives and constraints, adjusting their strategies dynamically based on observed outcomes and changing conditions. This dynamic approach is crucial for understanding the evolving nature of stakeholder interactions in TIPs. Unlike traditional game theory, which assumes rational actors with fixed strategies, EGM allows for bounded rationality, meaning participants adapt their behaviors over time based on perceived payoffs. This flexibility is crucial for TIPs, where stakeholders may alter their engagement depending on changing incentives, costs, or observed behaviors of others. TIPs require an approach that reflects the dynamic decision-making processes of participants, capturing shifts in response to incentives and penalties. EGM is uniquely suited to model this adaptive behavior, providing insights into how stakeholders evolve their strategies to maximize their benefits within the TIP framework. TIPs involve interdependent decision-making among manufacturers, recyclers, and consumers, with each group’s decisions affecting the others. EGM enables us to model these complex, multilateral interactions simultaneously, assessing how changes in one stakeholder’s strategy affect the others. By capturing the interdependencies among stakeholders, EGM provides a comprehensive framework for understanding TIP dynamics, unlike isolated or linear models that may overlook these interconnected behaviors. A core objective of this study is to identify conditions that lead to stable participation levels across stakeholders in TIPs. EGM allows for stability analysis through replicator dynamic equations, which reveal how different strategies evolve and stabilize over time. This analysis is essential to determine whether specific incentive structures or subsidy levels will foster sustained engagement among manufacturers, recyclers, and consumers. By examining these stable states, EGM offers predictive insights that can inform long-term TIP design and policy-making. EGM is highly adaptable to real-world scenarios, as it can incorporate various parameters such as subsidies, revenue-sharing ratios, and innovation costs. In this study, EGM accommodates factors like government subsidies, brand reputation, and environmental benefits, which directly impact stakeholder behavior in TIPs. This adaptability allows the model to reflect the realistic complexities of TIPs, making EGM a pragmatic choice for analyzing these multi-stakeholder environments. Through EGM, we can simulate various scenarios to observe how different initial conditions or parameter adjustments influence stakeholder strategies over time. This capability enables us to test the robustness of TIP strategies and make evidence-based recommendations for manufacturers and policymakers. The simulation approach enhances the practical relevance of our findings, as it provides a basis for decision-making under multiple potential market conditions.

## Problem description and modeling assumptions

### Problem description

**Fig 1:**
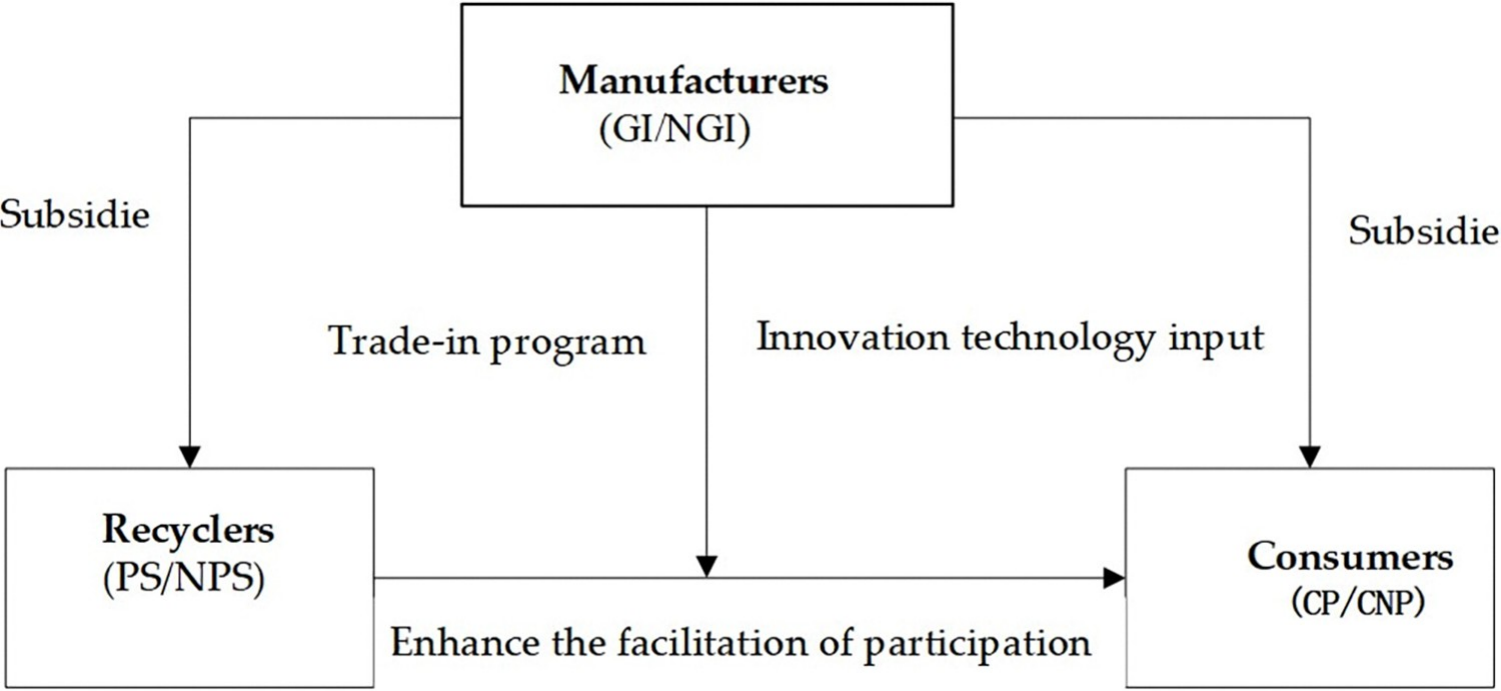
Relationship among the participants in TIP. (GI stands for manufacturers providing incentives to recyclers and customers; NGI stands for manufacturers choosing not to offer incentives; PS represents for participation strategy, NPS represents for non-participation; CP denotes that a customer will participate while CNP not participate in TIP).

This study examines a closed-loop recycling system where manufacturers, recyclers, and customers collaboratively participate in a TIP. by returning used products to manufacturers, while the TIP encourages customer and recycler involvement. An EGM is developed to explore the interdependence of these participants’ decisions and their sensitivities to different parameters within the supply chain. Fig 1 illustrates the interactions and relationships among these key stakeholders, highlighting the impacts of manufacturer incentives on decision-making and earnings across the supply chain.

### Modeling assumptions

In TIP, manufacturers face two strategic options: offering incentives (Option GI) with probability *x* or not offering incentives (Option NGI) with probability 1−*x*. Manufacturer revenue consists of three components: income from new products, recycling returns, and enhanced brand value, social responsibility, and government subsidies associated with TIP incentives, collectively denoted *ψ*_1_. If incentives are implemented, manufacturers may take three actions: invest in technological innovation (cost *T*), subsidize recyclers (subsidy *S*_1_) to encourage TIP participation, and directly incentivize customers (subsidy *S*_2_). The marketing and coordination costs for TIP are *C*_1_ with incentives and *C*_2_ without incentives.

Recyclers have two options: participate in TIP (Option PS) with probability *y*, or not participate (Option NPS) with probability 1−*y*. Their income includes recovery revenue, a share of new product sales revenue due to TIP participation, and any incentive subsidy *S*_1_ from manufacturers. Recyclers incur a cost *C*_3_ when participating in TIP.

Customers, as product sources for TIP, either participate (Option CP) with probability *z* or opt out (Option CNP) with probability 1−*z*. Participating customers gain a differential profit *K* from the new product’s use value minus the recycling value of the old product but incur an additional cost *C*_4_, representing convenience factors like communication and coordination in TIP.

Table 1 presents the definitions and explanations of the principal parameters and variables utilized in this study.

**Table 1:** Parameters and meaning.

Parameters	Meaning
*ψ*_1_ *ψ*_2_(*ψ*_3_) *R* *α* *β*	Revenue from brand reputation, social responsibility and government subsidies by incentive measures in TIP; Revenue from the sale of new products when customers participate (not participate) in TIP; Revenue from recycling of old products; Profit-sharing coefficient of manufacturers from the recycled product; Profit-sharing coefficient of manufacturers from the sale of new products;
*T* *C*_1_(*C*_2_) *C*_3_ *C*_4_	Cost of technological innovation when manufacturers choose incentives; Costs of marketing promotion and coordination with other enterprises for TIP conducted by manufacturers with and without incentives, respectively; Cost spent by recyclers when they participate in TIP; Cost incurred by the customers to participate in TIP without the participation of recyclers;
*S*_1_	Subsidie given by manufacturers to recyclers when they participate in TIP and manufacturers take incentive measures;
*S*_2_	Subsidie given by manufacturers to customers when they participate in TIP and manufacturers take incentive measures;
*K*	Profit for customers receive from participating in TIP;
*P*	Revenue from the old product for the customer when they don’t participate in TIP.

For EGM of the TIP, there are the following assumptions.

**Hypothesis 1:** If recyclers participate in TIP, the recycler facilitates product recovery to the manufacturer, generating a total revenue *R*, shared between manufacturers and recyclers as *αR* and (1−*α*)*R*, respectively.

**Hypothesis 2:** If both recyclers and customers participate, the revenue from new product sales, denoted *ψ*_2_, is divided between manufacturers, who receive *βψ*_2_, and recyclers, who receive (1−*β*)*ψ*_2_.
**Hypothesis 3:** If customers participate but recyclers do not, manufacturers still earn revenue *ψ*_2_ from new products and recycling revenue *R*, though with an additional recycling cost *d*. Without customer participation, new product revenue reduces to *ψ*_3_, reflecting decreased sales.
**Hypothesis 4:** Customers always dispose of old products. Participating customers gain a differential value *ψ*_3_ from new products, while non-participating customers receive recycling revenue *P* for their waste products collected by recyclers.

## Evolutionary game model and equilibrium analysis

### Income matrix in EGM

Table 2 presents the incomes of manufacturers, recyclers, and customers in the EGM. Each cell in the matrix reflects the income outcomes based on manufacturers adopting either the incentive strategy (GI) with probability *x* or no-incentive strategy (NGI) with probability 1−*x*, recyclers selecting participation (PS) with probability *y* or non-participation (NPS) with probability 1−*y*, and customers choosing to participate (CP) with probability *z* or not to participate (CNP) with probability 1−*z*.

**Table 2:** Income matrix of EGM in TIP.

Manufacturers	Recyclers	Customers	
		CP (*z*)	CNP(1−*z*)
**GI (*x*)**	**PS (*y*)**	${\psi_{1}+\beta \psi_{2}+\alpha R-C_{1}-T-S}_{1}-S_{2}$	${\psi_{1}+\psi }_{3}+\alpha R-C_{1}-S_{1}-T$
		$(1-\beta )\psi_{2}+(1-\alpha )R+S_{1}-C_{3}$	$(1-\alpha )R+S_{1}-C_{3}$
		*K*−*C*_4_+*S*_2_	P
	**NPS (1−*y*)**	$\psi_{1}+\psi_{2}+R-C_{1}-d-S_{2}-T$	$\psi_{1}+\psi_{3}+\alpha R-C_{1}-T$
		*0*	(1−α)R
		*K*−*C*_4_+*S*_2_	P
**NGI (1 – *x*)**	**PS (*y*)**	*βψ*_2_+*αR*−*C*_2_	$\psi_{3}+\alpha R-C_{2}$
		($(1-\beta )\psi_{2}+(1-\alpha )R-C_{3}$	$(1-\alpha )R-C_{3}$
		*K*−*C*_4_	P
	**NPS (1−*y*)**	*ψ*_2_+*R*−*d*−*C*_2_	$\psi_{3}+\alpha R-C_{2}$
		*0*	(1−α)R
		*K*−*C*_4_	P

### Analysis of tripartite game equilibrium

The replication dynamic for NFP is given as $F(x)=\frac{dx}{dt}$, where *x* denotes the probability that the NFP adopts DDM. Similarly, the replication dynamics for the carrier and shipper are $F(y)=\frac{dy}{dt}$ and $F(z)=\frac{dz}{dt}$, respectively.

#### Replication dynamic equation for manufacturers

If manufacturers opt to provide incentives in a TIP, the numerical equation for its anticipated return can be expressed as follows:

$$E_{11}=yz({\psi_{1}+\beta \psi_{2}+\alpha R-C_{1}-T-S}_{1}-S_{2})+y(\psi_{1}+\psi_{3}+\alpha R-T-C_{1}-S_{1})(1-z)+(1-y)(\psi_{1}+\psi_{2}+R-C_{1}-d-S_{2}-T)z+(1-y)(\psi_{1}+\psi_{3}+\alpha R-C_{1}-T)(1-z)$$ (1)

When manufacturers choose not to incentivize, the expected return is:

$$E_{12}=yz(\beta \psi_{2}+\alpha R-C_{2})+y(1-z)(\psi_{3}+\alpha R-C_{2})+(1-y)z(\psi_{2}+R-d-C_{2})+(1-y)(1-z)(\psi_{3}+\alpha R-C_{2})$$ (2)

The anticipated earnings of manufacturers are:

$$E_{1}=E_{11}x+E_{12}(1-x)$$ (3)

Thus, the replication dynamic equation under manufacturers’ GI tactics is:

$$F(x)=\frac{dx}{dt}=x(E_{11}-E_{1})=[C_{2}+\psi_{1}{-C}_{1}-T-S_{1}y-S_{2}z]x(1-x)$$ (4)

#### Replication dynamic equation for recyclers

If recyclers choose to participate in the TIP strategy, the expected return is:

$$E_{21}=xz((1-\beta )\psi_{2}+(1-\alpha )R+S_{1}-C_{3})+x(1-z)((1-\alpha )R+S_{1}-C_{3})+((1-\beta )\psi_{2}+(1-\alpha )R-C_{3})(1-x)z+((1-\alpha )R-C_{3})(1-x)(1-z)$$ (5)

When the recyclers choose not to participate in the TIP strategy, the expected return is:

$$E_{22}=x(1-z)(1-\alpha )R+(1-\alpha )R(1-x)(1-z)$$ (6)

The expected income of recyclers is:

$$E_{2}=E_{21}y+E_{22}(1-y)$$ (7)

The replication dynamic equation of recyclers with participate in TIPs is:

$$F(y)=\frac{dy}{dt}=y(E_{21}-E_{2})=[C_{3}-\psi_{2}z-S_{1}x-Rz+\beta \psi_{2}z+\alpha Rz]y(y-1)$$ (8)

#### Replication dynamic equation for customers

In the same way, when customers choose to participate in TIP, the expected return is:

$$E_{31}=xy({K-C_{4}+S}_{2})+x(1-y)({K-C_{4}+S}_{2})+(1-x)y(K-C_{4})+(1-x)(1-y)(K-C_{4})$$ (9)

When customers opt out of a TIP, the expected return is:

$$E_{32}=xyP+x(1-y)P+y(1-x)P+(1-y)(1-x)P$$ (10)

The expected income of customers is:

$$E_{3}=E_{31}z+E_{32}(1-z)$$ (11)

The dynamic equation of expectations for customers with participating in TIPs is:

$$F(z)=\frac{dz}{dt}=z(E_{31}-E_{3})=[C_{4}-K+P-S_{2}x]z(z-1)$$ (12)

The analysis of Formulas (4), (8), and (12) carries significant importance in advancing the understanding and investigation of the mechanisms and behaviors observed in dynamic replication systems, the foundational components of such systems. These formulas offer a precise mathematical representation of crucial parameters and variables inherent in dynamically replicating systems, thereby facilitating a more profound exploration of their intricate and evolving nature. The thorough examination of these formulas not only aids in uncovering latent principles and interaction mechanisms governing dynamically replicating systems but also bestows invaluable insights for predicting and optimizing system behaviors. Hence, delving into the intricacies of these formulas holds immense significance for the research and practical implementations of dynamic replication systems, thereby fostering advancements in associated academic domains.

### Jacobi matrix of the dynamical system

From the above dynamic replication system, several special stability points of the system can be acquired: P_1_ (0,0,0), P_2_ (1,0,0), P_3_ (0,1,0), P_4_ (0,0,1), P_5_ (1,1,0), P_6_ (1,0,1), P_7_ (0,1,1), and P_8_ (1,1,1). We carry out an in-depth analysis of the stability of these special points.

A Jacobian matrix is a matrix of partial derivatives that describes the slope or sensitivity of a vector function. We can construct the Jacobian matrix by taking the first partial derivatives of Eqs (4), (8) and (12) with respect to the variables x, y and z.


$$J=\left[\begin{array}{ccc} \frac{dF(x)}{d(x)} & \frac{dF(x)}{d(y)} & \frac{dF(x)}{d(z)}\\ \frac{dF(y)}{d(x)} & \frac{dF(y)}{d(y)} & \frac{dF(y)}{d(z)}\\ \frac{dF(z)}{d(x)} & \frac{dF(z)}{d(y)} & \frac{dF(z)}{d(z)} \end{array}\right]=\left[\begin{array}{ccc} J_{11} & J_{12} & J_{13}\\ J_{21} & J_{22} & J_{23}\\ J_{31} & J_{32} & J_{33} \end{array}\right]$$ (13)

$$J_{11}=(C_{2}+\psi_{1}{-C}_{1}-T-S_{1}y-S_{2}z)(1-2x)$$ (14)

$$J_{12}=-S_{1}x(1-x)$$ (15)

$$J_{13}=-S_{2}x(1-x)$$ (16)

$$J_{21}=S_{1}y(1-y)$$ (17)

$$J_{22}=[C_{3}-\psi_{2}z-S_{1}x-Rz+\beta \psi_{2}z+\alpha Rz](2y-1)$$ (18)

$$J_{23}=[\psi_{2}+R-\psi_{2}\beta -\alpha R]y(1-y)$$ (19)

$$J_{31}=S_{2}z(1-z)$$ (20)

$$J_{32}=0$$ (21)

$$J_{33}=(C_{4}-K+P-S_{2}x)(2z-1)$$ (22)

### Stability analysis

In the evolutionary game model, stability is assessed by evaluating the eigenvalues derived from the Jacobian matrix and eigenvalues derived from the system’s replication dynamic equations. Stability analysis reveals whether certain strategic decisions by manufacturers, recyclers, and customers will lead the system to a stable equilibrium, where all stakeholders benefit, or if it will diverge toward unstable behaviors. The sign of the eigenvalue determines the stability of the point. Stability occurs when the eigenvalues corresponding to the equilibrium points are negative, indicating that the system tends to return to equilibrium after small perturbations. The Jacobian matrix captures how each variable in the system—such as the strategic choices of manufacturers, recyclers and customers—affects the others. For instance, when the eigenvalues of an equilibrium point are all negative, the system is considered locally asymptotically stable. This means that any deviation from the equilibrium will decay over time, leading the system back to stability. Conversely, if one or more eigenvalues are positive, the system is unstable, and small deviations can drive the system away from equilibrium. Conditional stability arises when the eigenvalues are uncertain or include zero, indicating that the stability depends on specific conditions. By calculating the Jacobian matrix for each equilibrium point, we determined whether the system stabilizes when manufacturers adopt GI or when it refrains from doing so.

Each stable point represents a potential strategy profile where manufacturers may achieve optimal results depending on their goals, resources, and constraints. By identifying these stable configurations, manufacturers can make informed strategic decisions about TIP incentives, revenue-sharing, and other variables.

Through plugging in eight stability points into Jacobian matrix above, it can get the eigenvalues (*λ*_1_,*λ*_2_,*λ*_3_) and steady state which are presented in Table 3.

**Table 3:** Equilibrium points and their eigenvalues.

Equilibrium Points	Eigenvalue1s			Stability
	λ1	λ2	λ3	
(0,0,0)	-*C*_3_	*C*_2_−*C*_1_+*ψ*_1_−*T*	*K*−*C*_4_−*P*	Conditional stable
(1,0,0)	*S*_1_−*C*_3_	*C*_1_−*C*_2_+*ψ*_1_+*T*	*K*−*C*_4_−*P*+*S*_2_	Conditional stable
(0,1,0)	*C*_3_	*K*−*C*_4_−*P*	*C*_2_−*C*_1_+*ψ*_1_−*S*_1_−*T*	Unstable
(0,0,1)	*C*_4_−*K*+*P*	*C*_2_−*C*_1_−*ψ*_1_+*S*_2_−*T*	*ψ*2−*C*_3_+*R*−*βψ*_2_−αR	Conditional stable
(1,1,0)	*C*_3_−*S*_1_	*C*_1_−*C*_2_−*ψ*_1_+*S*_1_+*T*	*K*−*C*_4_−*P*+*S*_2_	Conditional stable
(1,0,1)	*C*_1_−*C*_2_−*ψ*_1_+*S*_2_+*T*	*C*_4_−*K*+*P*−*S*_2_	${\psi_{2}-C}_{3}+R+S_{1}-{\beta \psi }_{2}-\alpha R$	Conditional stable
(0,1,1)	*C*_4_−*K*+*P*	$C_{3}-\psi_{2}-R+{\beta \psi }_{2}+\alpha R$	$C_{2}-C_{1}+\psi_{1}-S_{1}-S_{2}-T$	Conditional stable
(1,1,1)	*C*_4_−*K*+*P*−*S*_2_	$C_{1}-C_{2}-\psi_{1}+S_{1}+S_{2}+T$	$C_{3}-\psi_{2}-R-S_{1}+{\beta \psi }_{2}+\alpha R$	Conditional stable

The stability of each equilibrium offers manufacturers critical insights into the most advantageous strategies for maximizing engagement in TIPs. For non-incentive Strategy P_1_ (0,0,0), because *λ*_1_ = −*C*_3_<0, and the other two eigenvalues are uncertain symbols. P_1_ (0,0,0) is conditional stable. When manufacturers choose not to provide incentives, recyclers do not participate, and customers choose not to engage, Stability at this point occurs when the cost difference between incentive and non-incentive strategies outweighs benefits from brand reputation or social responsibility. This scenario indicates that TIP incentives are impractical if costs are high relative to the gains, suggesting that manufacturers may avoid TIPs when subsidy costs are prohibitive or market response is weak.

For P_3_ (0,1,0), because *λ*_1_ = *C*_3_>0, P_3_ (0,1,0) is unstable. This suggests that recyclers face challenges in maintaining stable participation when manufacturers do not incentivize involvement and customers are unwilling to engage.

For the rest stability points, the corresponding eigenvalues are all not defined by positive or negative sign, and they are conditional stable points.

If *K*−*P*<*C*_4_ and *C*_1_+*T*−*C*_2_>*ψ*_1_ are fulfilled by the evolution system, the system can attain local stability. In this case, the stable strategy for the system is denoted as P_1_ (0,0,0). For manufacturers, *C*_1_+*T*−*C*_2_>*ψ*_1_ indicates that the cost difference between the manufacturers’ incentive and non-incentive is greater than the social reputation gained, in which case the manufacturers will choose no incentive measures. For customers, the advantages derived from engaging in TIP are outweighed by the associated costs, and customers of course choose not to participate in TIP.

When $K-P+S_{2}<C_{4},\psi_{1}>C_{1}+T+C_{2}$ and *S*_1_<*C*_3_ are fulfilled by the evolution system, the system can attain local stability. In this case, the stable strategy for the system is denoted as P_2_ (1,0,0). For manufacturers, *ψ*_1_>*C*_1_+*T*+*C*_2_ indicates that the social reputation and brand value gained by the manufacturers are greater than the sum of technological innovation investment and costs of the cooperation under the incentive and non-incentive conditions, and then the manufacturers will choose incentive measures. *S*_1_<*C*_3_ indicates that the recycling subsidy received by recyclers is smaller than the cost of participation, and recyclers tend not to participate in the cooperation at this time. *K*−*P*+*S*_2_<*C*_4_ indicates that in the case of recycling subsidies given by the manufacturers, the overall benefit of the customers is less than the participation cost, at this time, the customers of course choose not to participate in TIP.

When $K-P+S_{2}>C_{4},C_{1}+T+S_{2}-C_{2}>\psi_{1}$ and $(1-\beta )\psi_{2}+(1-\alpha )R<C_{3}$, in such a scenario, the optimal evolutionary strategy for the system can be identified as P_4_ (0,0,1). In this case, the cost difference between the manufacturers using incentive measure (excluding participation subsidies to recyclers) and not using incentive measures is larger than the revenue from brand reputation, social responsibility and government subsidies brought about by taking incentive measures in TIP, and the manufacturers are inclined to give no incentive. $(1-\beta )\psi_{2}+(1-\alpha )R<C_{3}$ indicates that when the recycler participates in the TIP, the benefit is less than the cost of his participation. From the economic point of view, the recyclers will certainly not participate in TIP. Under this circumstance, the total benefit of customers is greater than the participation cost, and customers tend to participate in TIP.

If $C_{3}<S_{1},C_{1}+T+S_{1}-C_{2}<\psi_{1}$ and *K*−*P*+*S*_2_<*C*_4_, in such a scenario, the optimal evolutionary strategy for the system can be identified as P_5_ (1,1,0). In this case, the cost difference between the manufacturer using incentive measures (excluding participation subsidies to customers) and the manufacturer not using incentive measures is lower than the revenue from brand reputation, social responsibility and government subsidies brought about by taking incentive measures in TIPs, and the manufacturers are inclined to give incentive. *C*_3_<*S*_1_ indicates that the participation cost of a recycler in the TIP is less than the subsidy he receives. From the perspective of pursuing profits, recyclers will certainly participate in TIPs. In this case, the total benefit of customers is lower than the participation cost, and customers tend to do not participate in the TIP. At this conditional stability point, manufacturers and recyclers participate while customers opt out. This suggests that without further subsidies or direct incentives for customers, TIPs may fail to reach full participation. Manufacturers could use this insight to balance the level of subsidies and other incentives to encourage broader engagement.

If $K-P+S_{2}>C_{4},\psi_{1}-T-S_{2}+C_{2}>C_{1}$ and $(1-\beta )\psi_{2}+(1-\alpha )R+S_{1}<C_{3}$, in such a scenario, the optimal evolutionary strategy for the system can be identified as P_6_ (1,0,1). In this case, the revenue from brand reputation, social responsibility and government subsidies brought about by taking incentive measures in TIPs is greater than the difference between the costs incurred by the incentive (excluding participation subsidies to recyclers) and the cost incurred when the incentive is not used, and the manufacturers are inclined to give incentive. In this case, for recyclers, the costs outweigh the benefits for PS. From the economic point of view, the recycler will certainly not participate in the TIP. But the total benefit of customers is larger than the participation cost, and customers tend to participating in the TIP.

If $K-P>C_{4},\psi_{1}<C_{1}+S_{1}+S_{2}+T-C_{2}$ and $(1-\beta )\psi_{2}+(1-\alpha )R>C_{3}$, in such a scenario, the optimal evolutionary strategy for the system can be identified as P_7_ (0,1,1). In this case, the cost difference between the manufacturer using incentive measure and not using incentive measures is larger than the revenue from brand reputation, social responsibility and government subsidies brought about by taking incentive measures in TIPs, and the manufacturer is inclined to give no incentive. $(1-\beta )\psi_{2}+(1-\alpha )R>C_{3}$ shows that the advantages derived from engaging in the TIP are outweighed by the associated costs. Under this circumstance, the recyclers will actively participate in TIP. *K*−*P*>*C*_4_ shows that the benefit of customers is greater than the cost of participation when there is no incentive from the manufacturer, and customers tend to participate in TIP.

If $K-P+S_{2}>C_{4},\psi_{1}>C_{1}+S_{1}+S_{2}+T-C_{2}$ and $(1-\beta )\psi_{2}+(1-\alpha )R+S_{1}>C_{3}$ are fulfilled by the evolution system, the system can attain local stability. In this case, the stable strategy for the system is denoted as P_8_(1,1,1). In this scenario, all players are fully engaged in the TIP—manufacturers provide incentives, recyclers participate, and customers trade in products. Stability is achieved here when the benefits from TIP incentives, such as brand enhancement, social responsibility, and government subsidies, exceed incentive costs. If the overall benefit of recyclers is greater than the cost of participation when there are subsidies from manufacturers, recyclers will choose to participate in the TIP. In the same way, the overall benefit of customers is greater than the participation cost when there are subsidies from manufacturers, and customers will choose to participate in the TIP. This finding suggests that manufacturers should leverage TIPs when government support or brand benefits are sufficient to cover costs, thereby fostering strong participation and a circular economy model. Considering the long-term development of the system and the maximization of the interests of each game participant, the equilibrium point is P_8_(1,1,1) is equal to the tripartite game players a useful strategy.

Based on the preceding analysis, it can be inferred that the EGM of TIPs decision management offers a theoretical framework for manufacturers, recyclers, and customers to make informed strategic choices. It is essential that the benefits derived from manufacturers’ incentives surpass the additional costs associated with these incentives. Furthermore, the advantages resulting from manufacturers’ incentives for recyclers and customers should outweigh their participation costs in the TIP, facilitating their interest and allowing the system to progress towards a stable strategy of incentivized participation. Moreover, manufacturers can establish a reasonable profit-sharing coefficient and appropriate subsidy levels to encourage the involvement of recyclers and customers. This will enhance their participation and contribute to the overall development of the system. In TIPs, recyclers play a critical role in facilitating product recovery, processing, and recycling. The decisions made by recyclers—whether to participate actively, adjust pricing structures, or partner closely with manufacturers—shape the availability and efficiency of recycling services, which directly influences the behavior of manufacturers and consumers. Consumer behavior plays a crucial role in shaping the overall system dynamics and directly influences the strategic decisions of manufacturers and recyclers. The decision of consumers to participate in TIPs affects the revenue and cost structures for manufacturers and recyclers, which in turn impacts their willingness to offer incentives or participate in recycling processes.

### Dynamics trend of manufacturers

According to equation (*x*) = 0, we can obtain the expression of the steady state dividing line. If $C_{2}+\psi_{1}{-C}_{1}-T-S_{1}y-S_{2}z=0$, i.e., $y=y^{*}=-\frac{C_{1}+T-C_{2}-\psi_{1}+S_{2}z}{S_{1}}$, then *F*(*x*) = = *F*′(*x*) = = 0, it indicates that the system is stable. In other words, if $y=y^{*}=-\frac{C_{1}+T-C_{2}-\psi_{1}+S_{2}z}{S_{1}}$, then manufacturers’ strategies tend to stabilize. The policy implemented by manufacturers has no direct influence on the direction of the system.

When $y>y^{*}=-\frac{C_{1}+T-C_{2}-\psi_{1}+S_{2}z}{S_{1}}$, combining Eqs (4) and (14), the system can be stabilized when x* = 1. In other words, if manufacturers opt to provide incentives to other members, the EGM is capable of achieving a steady state.

When $y<y^{*}=--\frac{C_{1}+T-C_{2}-\psi_{1}+S_{2}z}{S_{1}}$, combining Eqs (4) and (14), the system can be stabilized when x* = 0. In other words, if manufacturers don’t opt to provide incentives to other members, the EGM is capable of achieving a steady state.

**Fig 2:**
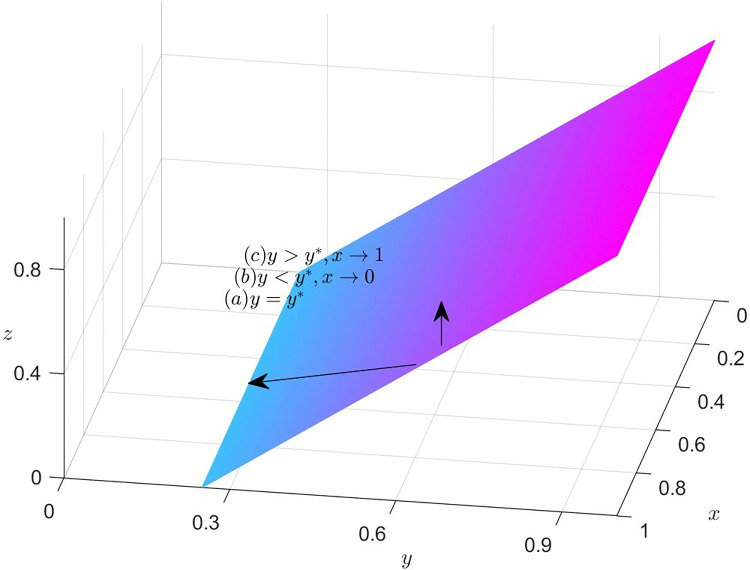
Dynamics trend of manufacturers.

Fig 2 depicts the decision-making dynamics evolution of manufacturers. Given that the joint space, defined by variables *y* and *z*, divides the feasible domain space into two adjacent parts, it is important to highlight the significance of this joint space by marking it in a distinguished color, such as purple. Meanwhile, the vendor’s mixed strategy space can be represented as the set of values for *x*, where *x* belongs to the interval [0,1]. To emphasize the trend of *x* within this range, an arrow can be utilized to indicate its direction. When it is in the lower part of the plane, *x* approaches 0 and is stable at 0. In this case, the manufacturer’s choice of non-incentive is optimal. When the system runs to the upper part of the plane, *x* approaches 1 and stabilizes at 1 point, then, the optimal strategy is for manufacturers to incentivize other members.

### Dynamics trend of recyclers

According to equation *F*(*y*) = 0, we can obtain the expression of the steady state dividing line. If $C_{3}-\psi_{2}z-S_{1}x-Rz+\beta \psi_{2}z+\alpha Rz=0$, i.e., $z=z^{*}=-\frac{C_{3}{-S}_{1}x}{\psi_{2}+R-\beta \psi_{2}-\alpha R}$, then *F*(*y*) = = *F*′(*y*) = = 0, it indicates that the system is stable. In other words, if $z=z^{*}=-\frac{C_{3}{-S}_{1}x}{\psi_{2}+R-\beta \psi_{2}-\alpha R}$, then recyclers’ strategies tend to stabilize. The choice implemented by recyclers has no direct influence on the direction of the system.

When $C_{3}-\psi_{2}z-S_{1}x-Rz+\beta \psi_{2}z+\alpha Rz\neq 0$, if *F*(*y*) = 0, then y* = 0, y* = 1. These are two stable points for recyclers.

When $z>z^{*}=-\frac{C_{3}{-S}_{1}x}{\psi_{2}+R-\beta \psi_{2}-\alpha R}$, combining Eqs (8) and (18), the system can be stabilized when y* = 1. In other words, if recyclers opt to participate in TIP, the EGM is capable of achieving a steady state.

When $z<z^{*}=-\frac{C_{3}{-S}_{1}x}{\psi_{2}+R-\beta \psi_{2}-\alpha R}$, combining Eqs (8) and (18), the system can be stabilized when y* = 0. In other words, if recyclers don’t opt to participate in TIP, the EGM is capable of achieving a steady state.

**Fig 3:**
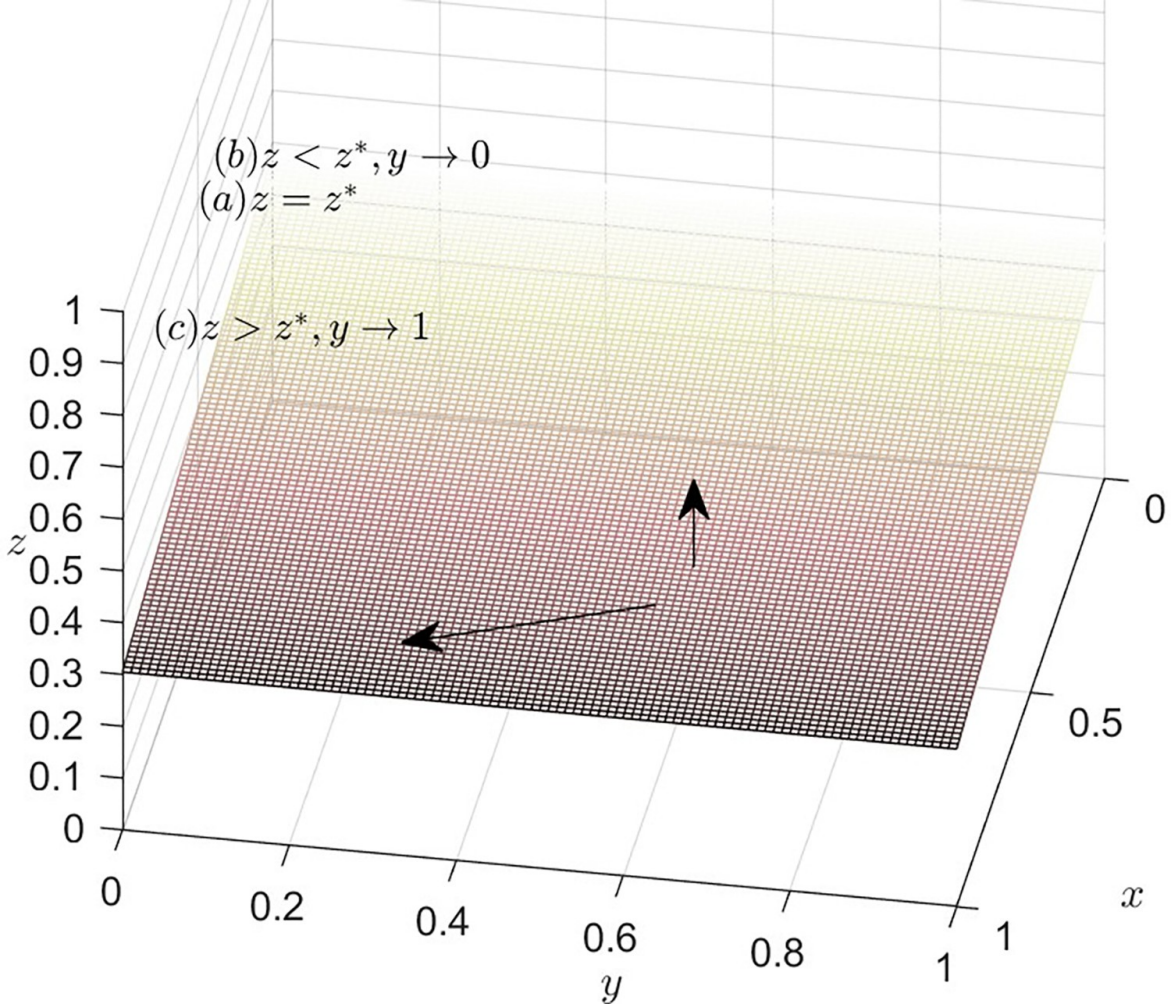
Dynamics trend of the recyclers.

Fig 3 depicts the decision-making dynamics evolution of recyclers. Given that the joint space, defined by variables *x* and *z*, divides the feasible domain space into two adjacent parts, it is important to highlight the significance of this joint space by marking it in a distinguished color, such as pink. Meanwhile, the vendor’s mixed strategy space can be represented as the set of values for *y*, where *y* belongs to the interval [0,1]. To emphasize the trend of *y* within this range, an arrow can be utilized to indicate its direction. When it is in the lower part of the plane, *y* approaches 0 and is stable at 0. In this case, the recyclers’ choice of NPS is optimal. When the system runs to the upper part of the plane, *y* approaches 1 and stabilizes at 1 point, then, the optimal strategy is for recyclers to choose PS.

### Dynamics trend of consumers

According to Formula (12), if *C*_4_−*K*+*P*−*S*_2_*x* = 0, i.e., $x=x^{*}=\frac{C_{4}-K+P}{S_{2}}$, then *F*(*z*) = = *F*′(*z*) = = 0, it indicates that the system is stable. In other words, if $x=x^{*}=\frac{C_{4}-K+P}{S_{2}}$, then consumers’ strategies tend to stabilize. The choice implemented by consumers has no direct influence on the direction of the system.

When *C*_4_−*K*+*P*−*S*_2_*x*≠0, if *F*(*z*) = 0, then z* = 0, z* = 1. These are two stable points for consumers.

When $x>x^{*}=\frac{C_{4}-K+P}{S_{2}}$, combining Eqs (12) and (22), the system can be stabilized when z* = 1. In other words, if consumers opt to participate in TIP, the EGM is capable of achieving a steady state.

When $x<x^{*}=\frac{C_{4}-K+P}{S_{2}}$, combining Eqs (12) and (22), the system can be stabilized when z* = 0. In other words, if consumers don’t opt to participate in TIP, the EGM is capable of achieving a steady state.

The change trend of the value of *z* is mainly divided into two different regions by the plane with fixed the value of *x*. That is to say, the choice of customers is mainly affected by the manufacturers, and little influenced by the choice of the recyclers. Because as long as the customer chooses to participate in TIP, if the recyclers participate in TIP, they can realize it through the recyclers; if the recyclers do not participate in, the manufacturers will adopt other ways to pay more recycling costs to realize the customer’s TIP.

According to the above analysis, whether manufacturers incentivize supply chain members, recyclers and consumers participate in TIP is closely related. Whether and in what way manufacturers incentivize supply chain members has a significant impact on whether recyclers and consumers participate in TIPs; meanwhile, whether recyclers and consumers participate in TIP behavioral decisions also influences manufacturers’ incentive actions and programs.

When recyclers actively participate in TIPs, they create a viable pathway for consumers to dispose of old products, making it easier and more convenient for consumers to participate. This increased availability of recycling services supports manufacturers by ensuring a reliable reverse logistics channel for collected products, reinforcing the recycling ecosystem. High recycler participation reduces the logistical burden on manufacturers, as recyclers handle collection and processing of traded-in products. This allows manufacturers to focus on product innovation and marketing rather than recycling logistics. Consequently, manufacturers may find it more feasible to offer subsidies or discounts to encourage consumer participate in TIPs, knowing recyclers are equipped to handle the inflow of products. A high level of recycler engagement encourages consumers to participate in TIPs, as accessible and reliable recycling services increase the appeal and convenience of trading in old products. When recyclers operate efficiently and offer convenient options, consumers are more likely to view TIPs as beneficial, supporting a higher rate of consumer engagement.

Consumer choices to engage in TIPs—by trading in used products for new ones—determine the volume of product returns and set the foundation for an effective recycling cycle. When consumers actively participate, manufacturers benefit from increased sales of new products and recyclers gain access to a steady stream of used items for processing. In contrast, low consumer participation can reduce product return rates, limiting recyclers’ revenue potential and disincentivizing manufacturers from offering further subsidies. High consumer participation creates strong demand for manufacturers’ new products and enhances brand value by supporting sustainable practices. This encourages manufacturers to allocate more resources to TIP incentives, such as discounts or subsidies, to maintain high engagement. Conversely, low consumer participation discourages manufacturers from investing in TIPs, as the lack of consumer buy-in fails to justify incentive costs. A strong consumer response increases the volume of recyclable goods, making TIPs more attractive for recyclers. With higher product flow, recyclers can achieve economies of scale, reducing per-unit recycling costs and increasing profitability. In cases of low consumer participation, recyclers may lack sufficient volume to justify participation, especially if their costs exceed potential revenue from processed products.

## Simulation

To intuitively observe the dynamic evolution of stakeholders’ strategy, a numerical simulation is made to further investigate the decision-making influence of manufacturers, recyclers, and consumers by MATLAB software. The dataset for this research was compiled from real interactions within the TIP environment, sampling a diverse group of stakeholders to capture a full range of behaviors and decision-making processes. It includes both quantitative and qualitative data from manufacturers, recyclers, and consumers, providing a solid foundation for analyzing stakeholder behavior. Key variables such as incentive structures, recycling costs, consumer participation rates, and subsidy levels offer a multi-dimensional view of the factors influencing strategic decisions in TIPs.

Numerical simulations were conducted to test various market scenarios, focusing on how different initial conditions and key parameters—such as subsidy levels, penalties, and incentive costs and revenue**s**—influence stakeholder behavior. The model is assigned numerical values according to the actual situation. It is assumed that array 1: $\alpha =0.7,\;\beta =0.8,\;K=100,\;R=150,\;P=20,\;T=80,\;\psi_{1}=150,\;{\psi_{2}=350,\;S}_{1}=20,\;S_{2}=15,\;C_{1}=25,\;C_{2}=10,\;C_{3}=55,\;C_{4}=5.$

### The impact of the initial value on EGM

**Fig 4:**
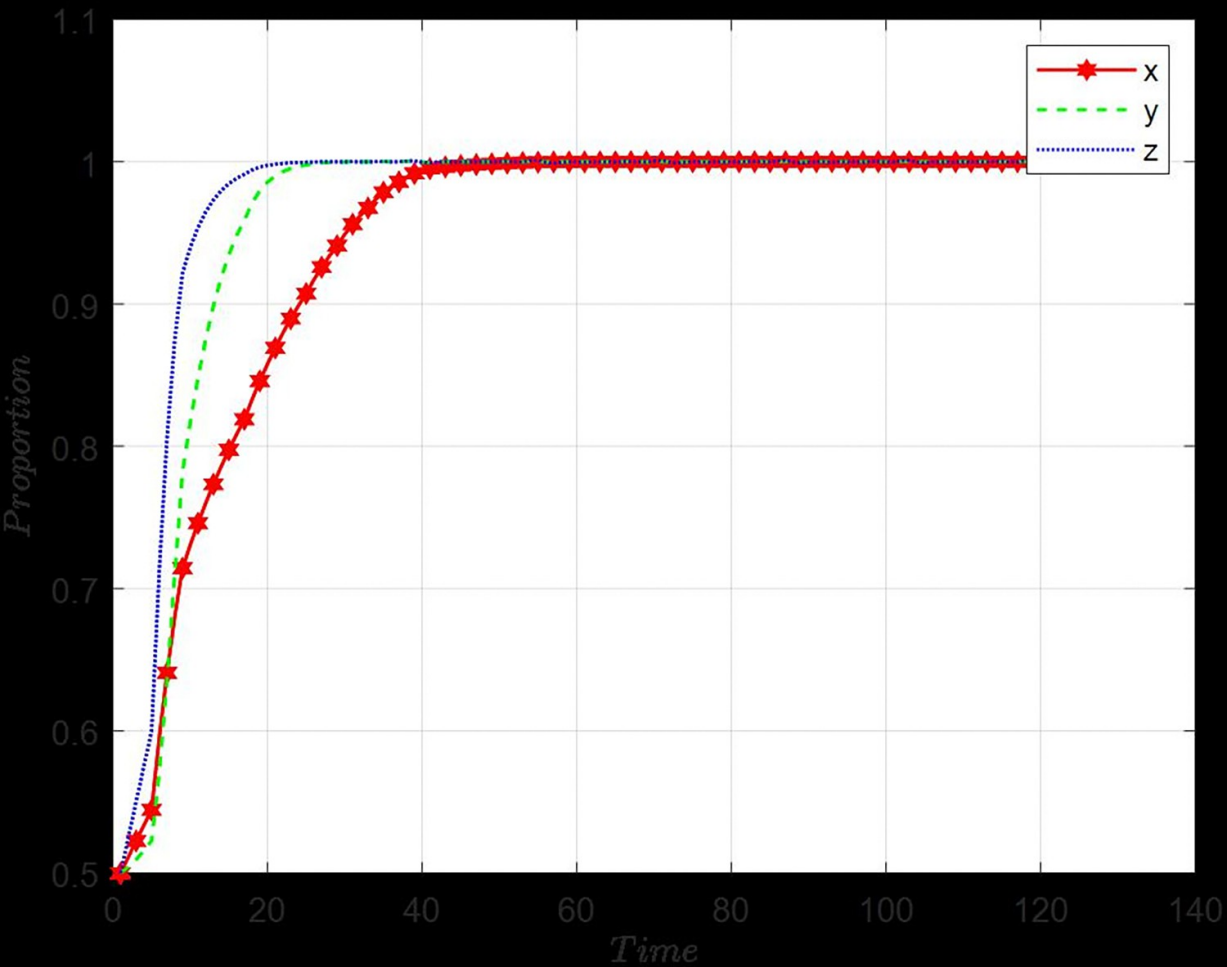
Effect of initial setting.

Let *x*_0_,*y*_0_ and *z*_0_ are the initial points of *x*,*y* and *z*, respectively. Here, setting *x*_0_ = *y*_0_ = *z*_0_ = 0.5. The following section will explore the influence of various starting points on the choice patterns of the three entities. Fig 4 shows that from the initial point, how do manufacturers, recyclers, and customers dynamically evolve toward the stable point (GI, PS, CP). If consumers choose CP, recyclers evaluate consumers’ behavior and choose PS. In the context of the market, it is not surprising that manufacturers’ decision-making processes regarding recycling fall behind those of recyclers and consumers.

### The impact of the initial probability on EGM

**Fig 5:**
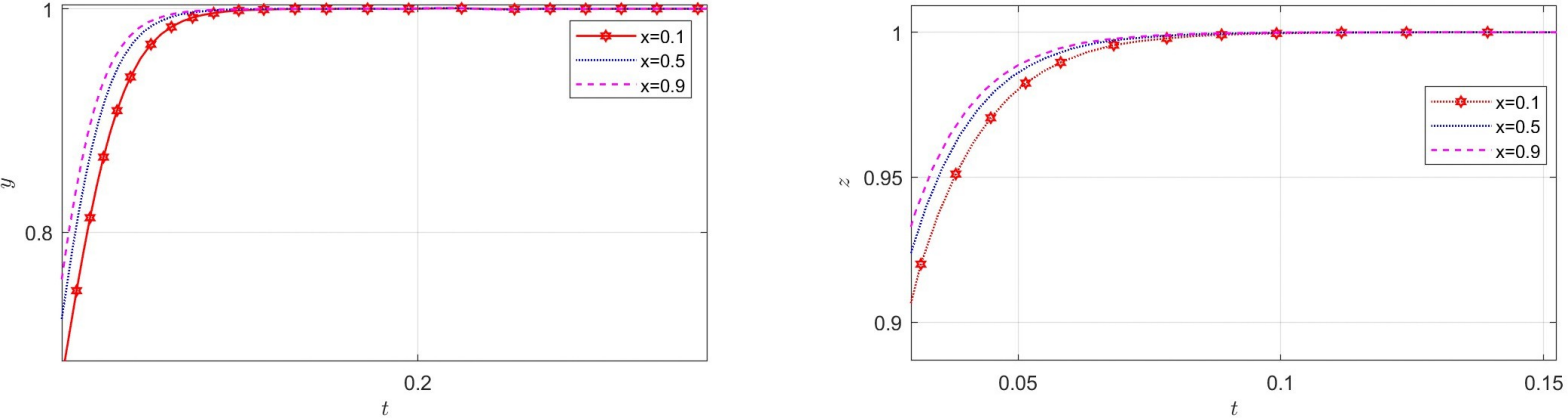
The impact of initial probability of manufacturers on recyclers and consumers.

**Fig 6:**
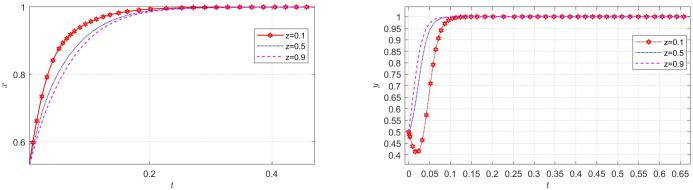
The impact of initial probability of recyclers on manufacturers and consumers.

**Fig 7:**
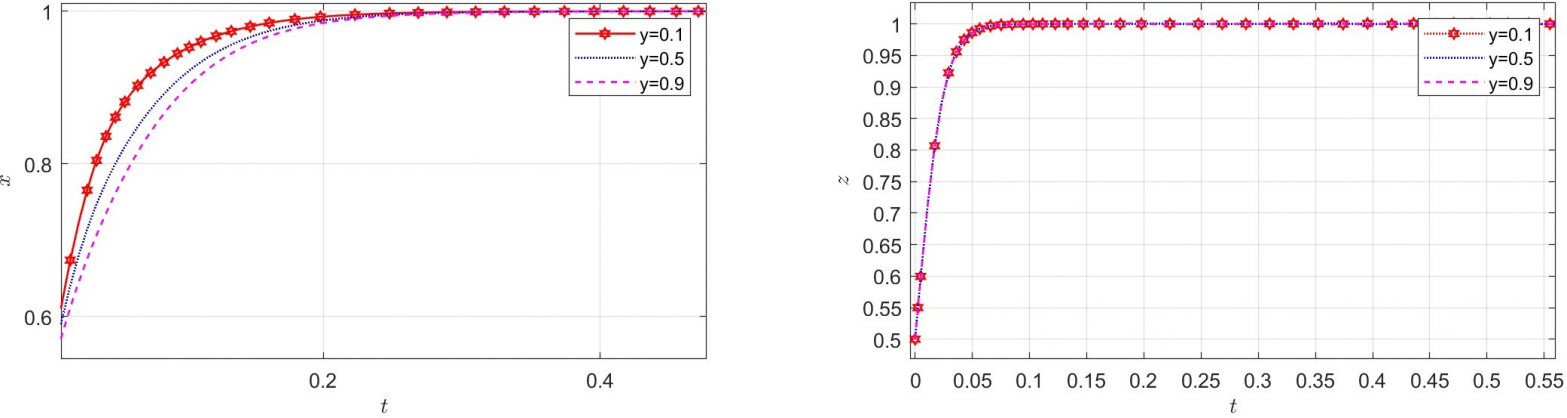
The impact of initial probability of consumers on manufacturers and recyclers.

This section discusses the influence of the initial value selected by each player on the equilibrium under other factors unchanged. The outcomes are illustrated in Figs 5–7.

Fig 5 illustrates how the initial probability of manufacturers choosing GI influences the evolution of recyclers and consumers strategies. The diagram located to the left in Fig 5 shows that the probability that the manufacturers choose GI increases, recyclers choose PS faster, but the growth is not obvious, the promotion effect on recyclers is smaller. Therefore, manufacturers should increase the incentive ratio at the early stage, giving recyclers positive signals to encourage them to participate in the TIP, rather than supporting the decision later. The diagram located to the right in Fig 5 shows that as the probability of GI strategy of a manufacturer increases, an increase in consumer choice of CP is observed. But the change has not been as dramatic as for recyclers. In the actual setting, the impact of manufacturers’ decisions on consumers is constrained by the decisions made by recyclers, thereby attenuating the influence of manufacturers’ behaviors on consumers.

Fig 6 shows how the initial probability of recyclers choosing PS affects the evolution of manufacturers and customers’ strategies. According to the diagram located to the left in Fig 6, The longer it takes for the manufacturer to adopt the GI strategy and stabilize it, the higher the probability that the recyclers will choose PS. Consequently, in trade-in market with poor performance, manufacturers are more inclined to strategically intervene at the optimal time. It can be seen from the diagram located to the right in Fig 6 that the choice of consumers does not fluctuate significantly due to the change of the choice of recyclers. Because manufacturers’ incentives and additional recycling costs reduce customers’ reliance on recyclers.

The initial probability of customers choosing CP strategy has opposite effects on manufacturers and recyclers. Fig 7 illustrates how the initial probability of customers choosing CP strategy affects the evolution of manufacturers and recyclers’ strategies. The diagram located to the left in Fig 7 shows that as the probability of CP strategy of customers increases, it is observed that the more difficult it is for manufacturers to achieve GI policy stability. It shows that for manufacturers, the lower the initial probability of customers choosing to CP, the more the manufacturers need to intervene to influence the market, so the manufacturers are more eager to use GI methods to motivate consumers and recyclers. It can be seen from the diagram located to the right in Fig 7 that recyclers will choose PS more quickly and achieve stability in this choice when it is observed that customers choosing CP with greater probability. This is in line with the reality. In reality, recyclers need to observe manufacturers’ measures and market reactions to decide whether to participate in TIPs.

### The impact of parameters on EGM

**Fig 8:**
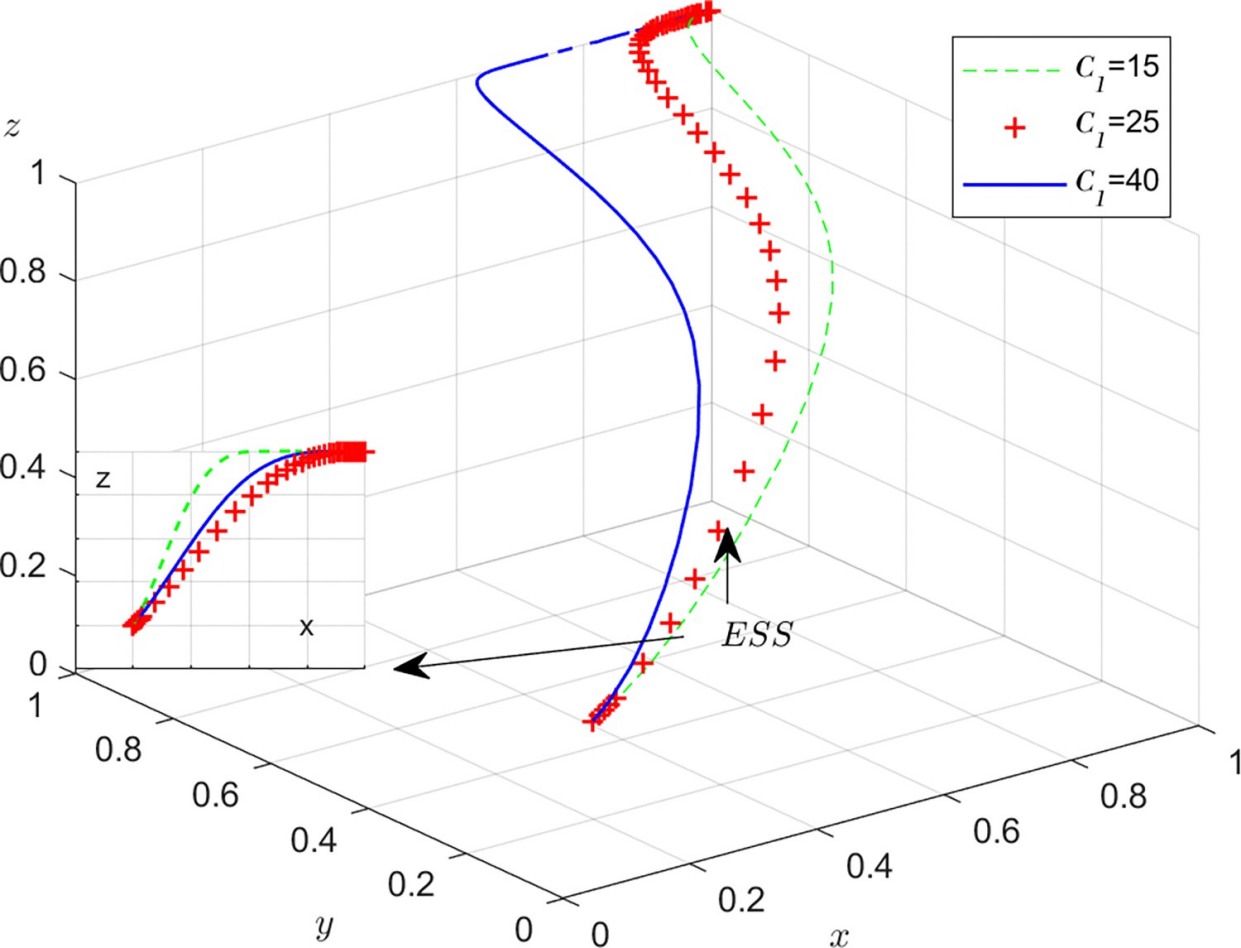
The influence of the incentives cost *C*_1_.

**Fig 9:**
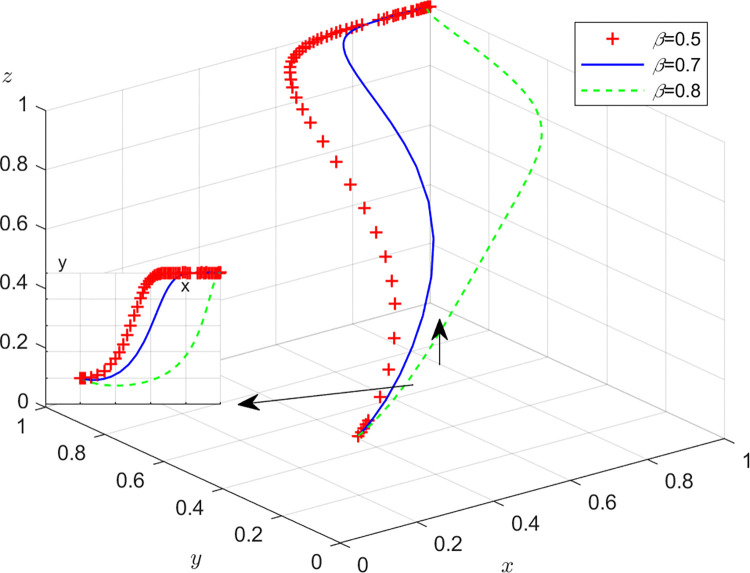
The influence of recycling revenue sharing ratio *α*.

**Fig 10:**
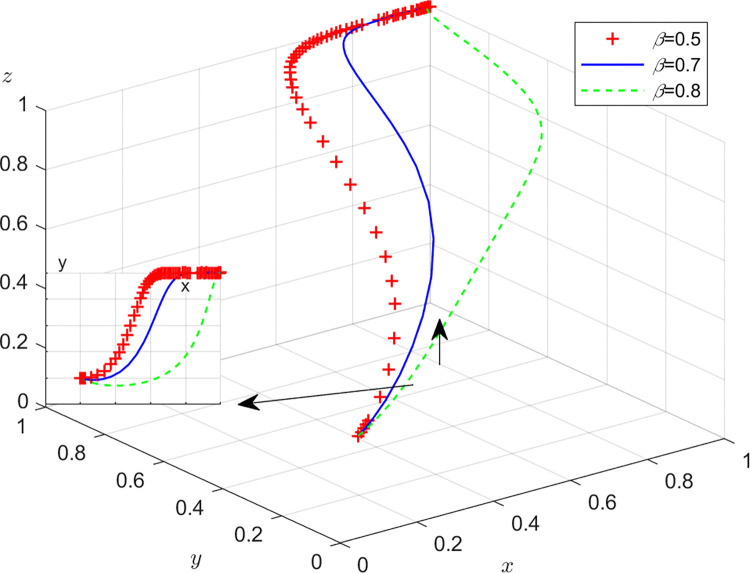
The influence of revenue sharing ratio *β*.

**Fig 11:**
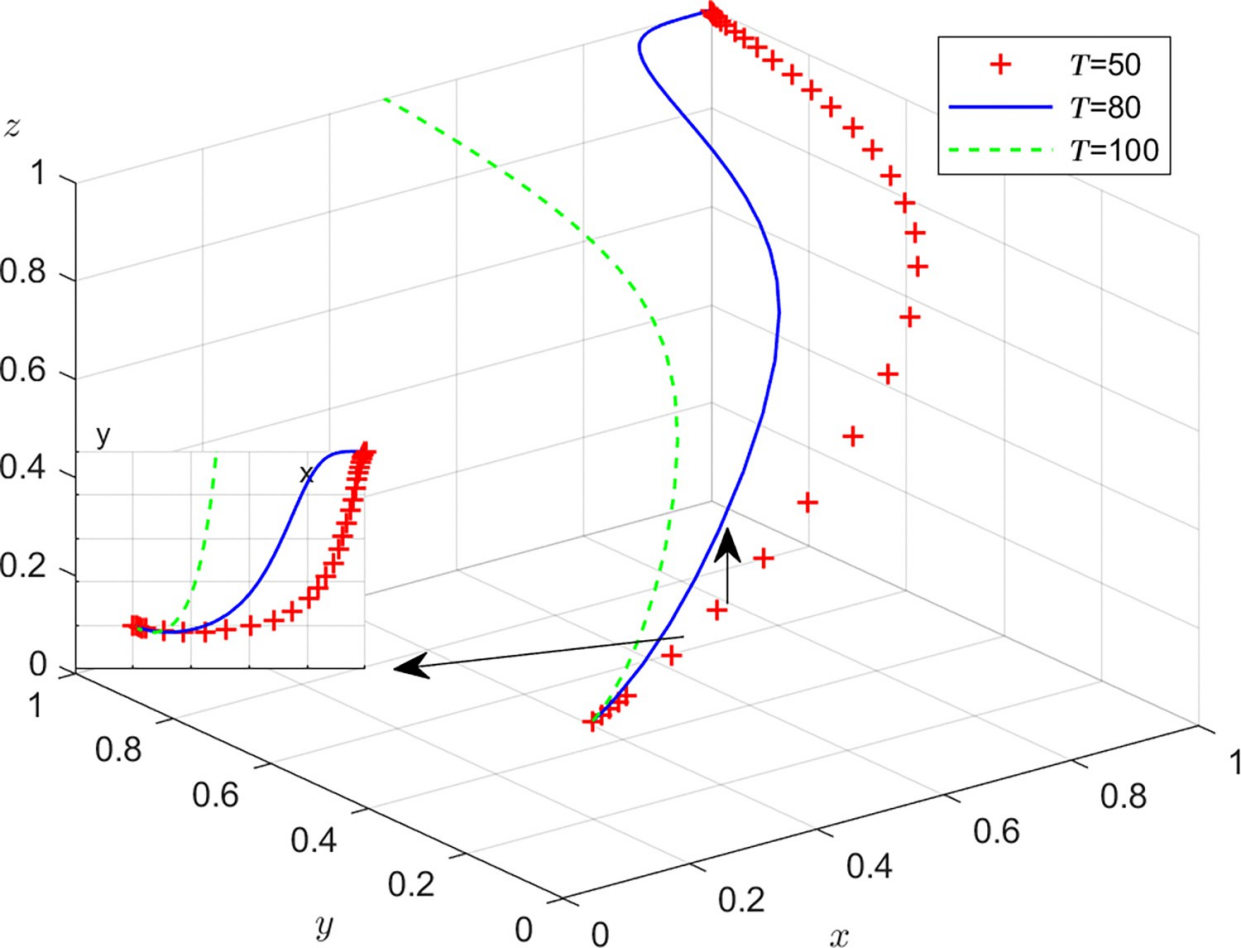
The influence of innovation input cost *T* for manufacturers.

**Fig 12:**
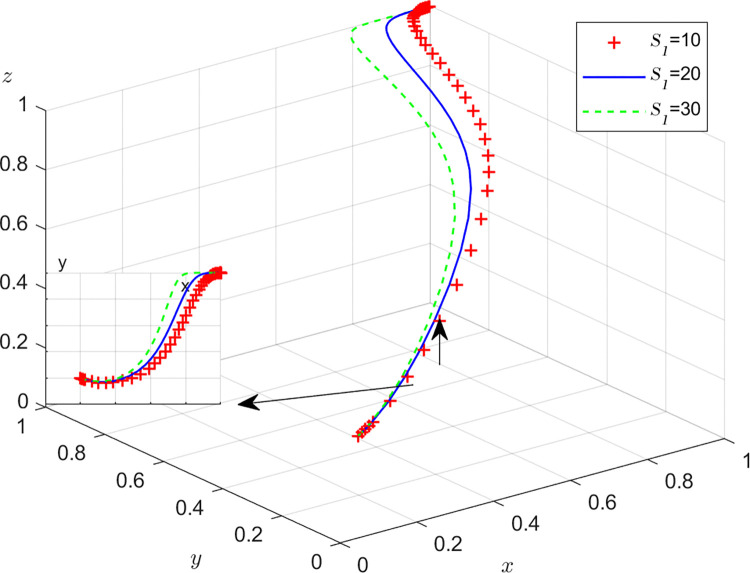
The influence of subsidie *S*_2_ of recyclers from manufacturers.

**Fig 13:**
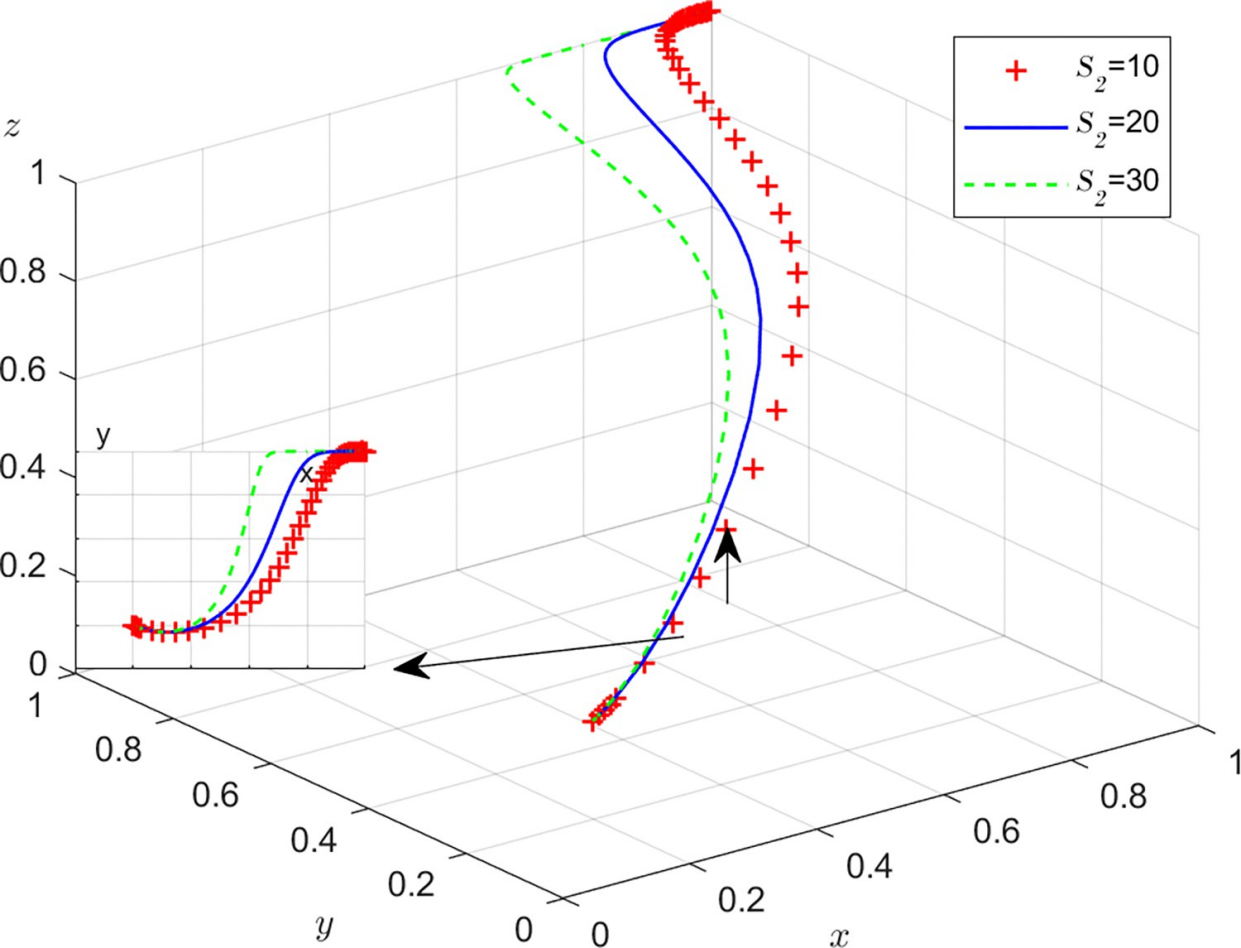
The influence of subsidie *S*_2_ of customers from manufacturers.

**Fig 14:**
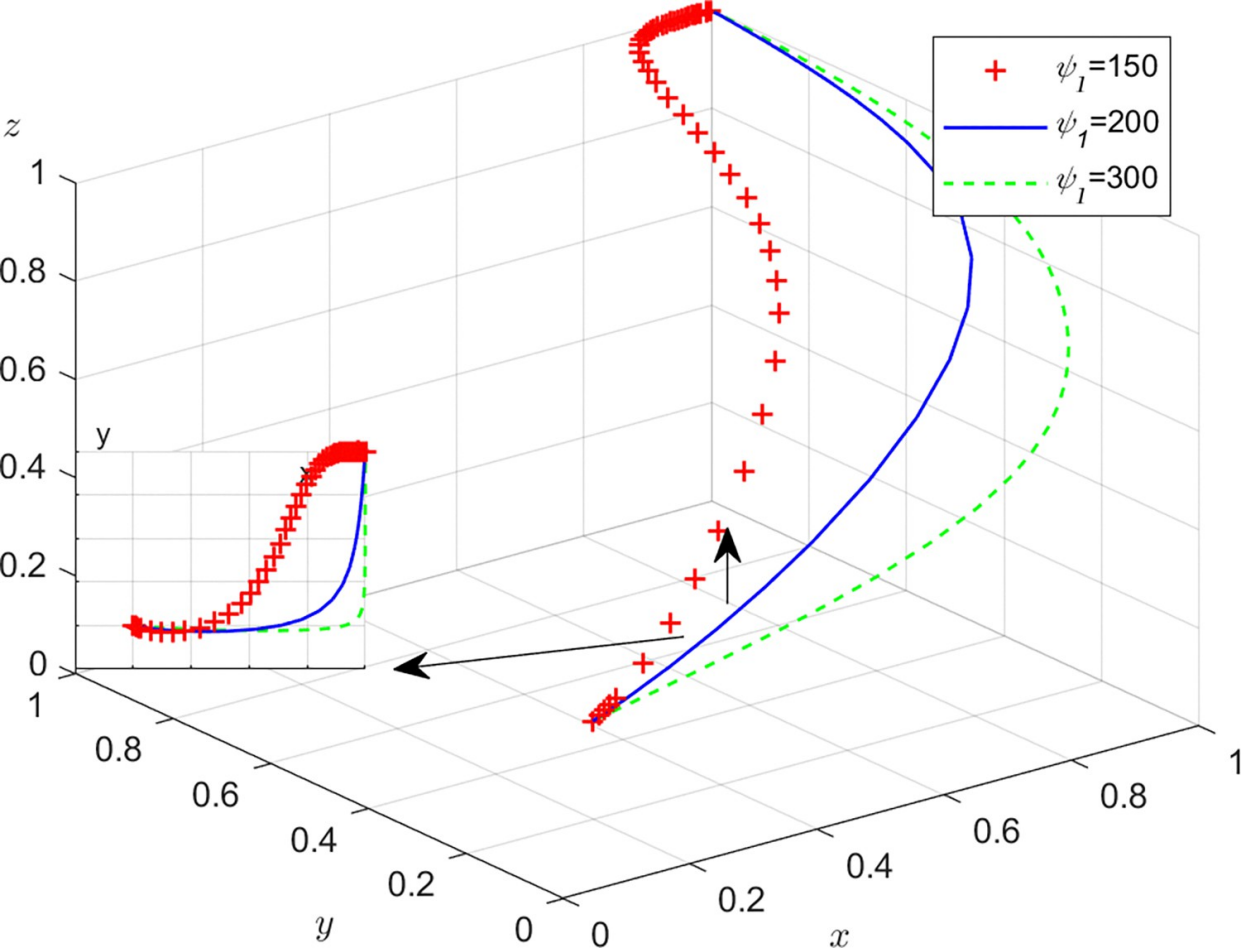
The influence of social and brand benefit *ψ*_1_ from GI.

Aiming at achieving a more profound comprehension of the influence of parameters on the participants, this study investigates several conditions pertaining to the five parameters encompassed within the comprehensive EGM. These parameters are systematically categorized into five distinct scenarios, enabling a comprehensive examination of their respective effects. First, for analyzing that how *C*_1_ affects the process and results of evolutionary game, *C*_1_ was assigned *C*_1_ = 15,25,40, and the simulation results of 50 times evolution of dynamic equations over time were replicated, as shown in Fig 8. In order to analyze the influence of *α* and *β*,*α* and *β* are assigned 0.5,0.7,0.8, respectively. Figs 9 and 10 show the simulation trends. In order to analyze the influence of *T*,*T* is assigned *T* = 50,80,100 respectively, and Fig 11 shows the simulation trends. For analyzing the influence of *S*_1_,*S*_2_, they are assigned 10,20,30 respectively. We can see the simulation results from Figs 12 and 13. In order to analyze the influence of *ψ*_1_,*ψ*_1_ is assigned *ψ*_1_ = 150,200,300 respectively, and Fig 14 shows the simulation trends.

#### Scenario 1. The influence of manufacturers’ incentive costs *C*_1_

Fig 8 shows how manufacturers’ incentive costs *R*_1_ influences the strategies of the system. According to Fig 8, with the increase of incentive cost *C*_1_, the probability of manufacturers choosing GI decreases, however, the probabilities of recyclers choosing PS and customers choosing CP are increasing rapidly. The increase of *C*_1_ reduces manufacturers’ desire to adopt incentive measures. However, the increase in incentive coordination costs of manufacturers will bring better service to recyclers and customers, thus accelerating the participation of recyclers and customers in TIPs.

#### Scenario 2. The influence of revenue-sharing coefficients *α* and *β*

Figs 9 and 10 show how revenue-sharing coefficients *α* and *β* affect the strategies of manufacturers, recyclers and customers. According to Figs 9 and 10, with the increase of recycling revenue sharing coefficients *α*,*β*, respectively, the probability of manufacturers with GI is accelerating, however, the probabilities of recyclers with PS and customers choosing CP are declining rapidly. The increase of *α* can increase the aggregate income of manufacturers and reduce the aggregate income of recyclers. Of course, manufacturers are more inclined to choose incentives, while recyclers will choose not to participate. The choice of customers is influenced by manufacturers and recyclers, and the probability of choosing CP is also decreasing.

#### Scenario 3. The influence of innovation input cost *T* for manufacturers

Fig 11 shows how innovation input cost *T* for manufacturers affect the strategies of manufacturers, recyclers and customers. According to Fig 11, with the increase of innovation input cost *T* for manufacturers, the probability of manufacturers choosing GI is first closer to 1, and then closer to 0 when *T* reaches a certain value, the probabilities of recyclers choosing PS and customers choosing CP are accelerating towards 1. This is consistent with the reality. At the beginning, with the increase of *T*, the appeal of the new product will be greatly enhanced, but the participation of recyclers and customers is not very high. At this time, if the manufacturers choose GI, the probabilities of recyclers and customers participating in the TIP will increase rapidly. However, if the innovation input cost is much higher and the market is relatively good, the manufacturer will choose NGI to reduce the cost. Innovation investment *T* can help manufacturers improve competitiveness, open new markets, improve productivity and profitability. It is not only the embodiment of enterprise competitiveness, but also an important way of social responsibility and sustainable development.

#### Scenario 4. The influence of subsidies *S*_1_,*S*_2_ from manufacturers

Figs 12 and 13 show how subsidies *S*_1_,*S*_2_ from manufacturers affect the strategies of manufacturers,recyclers and customers. As can be seen from Figs 12 and 13, *S*_1_ and *S*_2_ have the same impact on the system. In the process of system evolution, with the increase of subsidies *S*_1_,*S*_2_ from manufacturers, the probability of manufacturers choosing GI is first closer to 0, the probabilities of recyclers choosing PS and customers choosing CP are accelerating towards 1. The more subsidies, the greater the cost of manufacturers, the more reluctant to choose GI, and the increase in subsidies has a positive effect on recyclers and customers. Manufacturers should reasonably control the intensity of subsidies and find a balance between revenue and social responsibility.

#### Scenario 5. The influence of benefit *ψ*_1_ for manufacturers after adopting GI

Fig 14 show how benefit *ψ*_1_ for manufacturers after adopting GI affect the strategies of manufacturers, recyclers and customers. According to Fig 14, with the increase of the revenue *ψ*_1_ from brand reputation, social responsibility and government subsidies brought about by taking incentive measures in TIPs for manufacturers after adopting GI, the probabilities of manufacturers choosing GI, recyclers choosing PS and customers choosing CP are accelerating towards 1. At the same time, it can be seen that *ψ*_1_ has greater impact on manufacturers than recyclers and customers. This is consistent with the reality. *ψ*_1_ has a direct influence on manufacturers and an indirect impact on business members. Manufacturers should work closely with the government and strive for more support from the government. At the same time, they should promote their brands in the TIP, demonstrate their social responsibility, and improve their social image and brand influence.

Analysis from above, Consumers and manufacturers are more likely to participate in TIPs if recyclers are known to be reliable and trustworthy. Recyclers with strong reputations for environmental responsibility and efficient processing can strengthen consumer confidence in TIPs, encouraging higher participation rates. From a manufacturer’s perspective, working with reputable recyclers aligns with brand goals and minimizes risk.

Consumer behavior is highly responsive to incentives, such as price discounts or subsidies, which manufacturers offer to encourage TIP participation. Consumers’ decisions are shaped by the perceived value of these incentives, their interest in new product features, and the ease of the TIP. This sensitivity to incentives requires manufacturers to carefully balance the incentive levels to maximize engagement while controlling costs. Consumers’ incentive sensitivity pushes manufacturers to optimize their subsidy allocations. Offering adequate discounts encourages consumers to trade in outdated products, boosting sales of newer models. However, excessive incentives may erode manufacturers’ profit margins. By analyzing consumer response to different levels of incentives, manufacturers can determine the optimal balance between consumer engagement and profitability. Higher consumer participation, driven by effective manufacturer incentives, indirectly benefits recyclers by ensuring a consistent supply of used products. When consumers are more willing to trade in their products, recyclers can invest confidently in recycling infrastructure and partnerships. This enables recyclers to operate efficiently and meet demand, creating a stable supply chain loop.

Consumers are more likely to participate in TIPs when they perceive brands as environmentally responsible and trustworthy. Positive brand perception enhances consumer engagement, which in turn supports manufacturers’ long-term strategies. Brand loyalty among environmentally conscious consumers can lead to a more stable and predictable participation rate, supporting continuous TIP engagement. Building consumer trust and enhancing brand reputation through TIPs encourages sustained participation. Consumers who view TIPs as an effective way to support sustainable practices are more likely to engage, increasing overall TIP effectiveness. Consequently, manufacturers can reduce the reliance on financial incentives over time as consumer loyalty strengthens, making TIPs more cost-effective. For recyclers, a trusted brand association with TIPs ensures a reliable product inflow. When consumers associate TIPs with environmental responsibility, recyclers benefit from a steady supply of products, even with minimal direct marketing efforts. Thus, recyclers are more likely to collaborate with manufacturers that have strong brand credibility, as this partnership enhances recyclers’ own brand reputation and operational stability.

**Fig 15:**
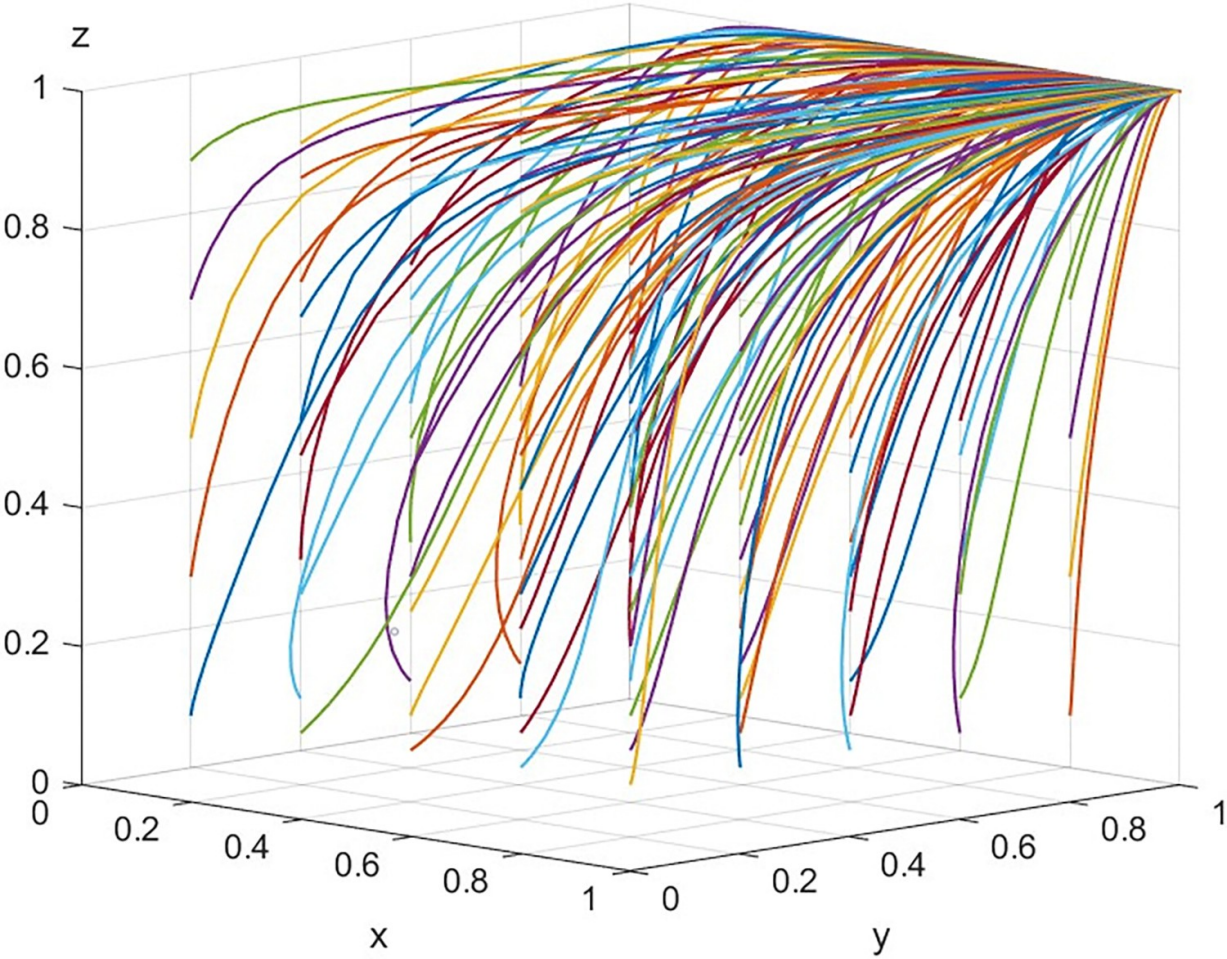
The result of 50 evolutions of array 1.

Next, we will consider that array 1 evolves 50 times at different times with different initial policy combinations. Fig 15 shows the results of evolution.

According to Fig 15, the simulation results show that there is only one evolutionarily stable strategy combination in the system at this time P_8_ (1,1,1), that is (GI, PS, and CP), which statement aligns with the findings of the asymptotic stability analysis presented in the stability analysis section.

In TIPs, manufacturers play a central role by determining incentives, coordinating with recyclers, and marketing TIPs to consumers. Their strategies and decisions significantly influence both the behavior of recyclers and the level of consumer engagement, which together define the effectiveness and stability of TIPs. Through carefully managed incentives, revenue-sharing agreements, sustainable product designs, and brand reputation, manufacturers create a supportive environment that maximizes recycler and consumer engagement. By aligning their actions with the interests of recyclers and consumers, manufacturers can foster a resilient, sustainable TIP model that promotes environmental responsibility, supports a circular economy, and delivers lasting benefits to all stakeholders involved.

Recyclers help stabilize the TIP ecosystem by providing a consistent channel for old products, ensuring that the flow of trade-ins remains steady. This stability encourages manufacturers to sustain TIP incentives, as a dependable recycling system supports both the circular economy model and manufacturers’ strategic objectives. Consumer behavior influences not only the quantity of traded-in products but also the strategic equilibrium of the system. For instance, if consumers exhibit low trade-in rates, the TIP system may shift towards an unstable state where manufacturers reduce incentives and recyclers lower participation. Conversely, high consumer engagement can push the system toward stable, full participation, where manufacturers maintain incentives, and recyclers consistently process returned products. High levels of consumer participation contribute to equilibrium by stabilizing the strategies of manufacturers and recyclers. This full-participation equilibrium benefits the system by optimizing product flows, increasing recycling rates, and enhancing sustainability. In a scenario where consumer participation is unstable, manufacturers may need to adjust their strategies by offering higher subsidies or partnering more closely with recyclers to maintain stability. To adapt to consumer behavior trends, manufacturers and recyclers need to remain flexible in their approach to TIPs. For example, if consumer interest wanes, manufacturers might introduce limited-time promotions or additional loyalty incentives to stimulate engagement. Recyclers can also adjust their processing capacities based on fluctuations in consumer behavior, scaling operations as consumer participation grows.

The simulation analysis demonstrates a high level of consistency, aligning with scholarly standards. It is effective with the conclusion of the strategic stability analysis of all parties, and has practical guiding significance for TIPs of manufacturer-led.

## Conclusions

This study provides an in-depth analysis of the dynamics of TIPs within a closed-loop recycling system, utilizing an EGM to examine how manufacturers, recyclers, and customers strategically adapt their decisions in response to incentives, costs, and potential gains. Our findings emphasize the importance of strategic subsidies, revenue-sharing structures, and innovation investments in promoting TIP effectiveness and advancing sustainability in recycling. The findings reveal actionable insights for industry practitioners and policymakers aiming to enhance the effectiveness and sustainability of TIPs.

Manufacturer-provided subsidies are shown to be a critical factor in achieving system equilibrium by encouraging recyclers and customers engagement in TIPs. Higher subsidies directly increase participation rates among these stakeholders. This study supports the argument for well-designed subsidy structures as integral to TIP stability and highlights the role of subsidies as an effective tool for optimizing stakeholder engagement. This suggests that manufacturers and policymakers should prioritize well-structured subsidy frameworks that target both stakeholders to maximize TIP engagement. In practice, manufacturers could implement tiered subsidy programs that adjust incentives based on recyclers and customers’ commitment levels. For policymakers, incentivizing manufacturers to provide subsidies could enhance overall participation, creating a more sustainable recycling ecosystem. Manufacturers should design flexible, targeted subsidies that encourage high engagement across TIP stakeholders, while policymakers could support these efforts by offering tax incentives or subsidies to manufacturers that promote recycling through TIPs. This finding provides actionable insights for manufacturers and policymakers, showing that strategic subsidy allocation can strengthen participation and system sustainability. It contributes to the body of knowledge by quantifying how subsidies directly impact TIP stability, supporting sustainable recycling practices.

The study finds that revenue-sharing ratios significantly impact the system’s stability by influencing recycler and customer participation. While lower revenue-sharing ratios may reduce immediate returns for manufacturers, they create a stronger incentive for recycler participation, which in turn positively influences customer engagement. This finding suggests that manufacturers need to adopt a balanced revenue-sharing approach, considering both short-term revenue impacts and the long-term advantages of an engaged recycling network. Manufacturers should adopt a revenue-sharing framework that aligns with their sustainability goals. For instance, they could implement lower revenue-sharing ratios initially to attract recyclers, then gradually increase shares as the recycling network stabilizes and grows. By clarifying how revenue-sharing structures affect TIP dynamics, this conclusion provides manufacturers with a strategic framework to manage incentives, which supports operational stability and fosters sustainable recycling ecosystems.

High costs associated with incentive programs and technological innovation can discourage manufacturers from investing in TIPs. These findings indicate that manufacturers should carefully assess these costs against expected increases in participation rates. For real-world application, manufacturers could adopt cost-sharing strategies by collaborating with government agencies or recycling firms to distribute the financial burden of innovation and incentive costs. Moreover, prioritizing cost-effective innovations that reduce TIP expenses can make the system more financially sustainable. Manufacturers should seek partnerships or government subsidies for cost-intensive initiatives, such as innovation investments, to reduce financial strain while maintaining TIP efficiency. Additionally, adopting incremental innovation strategies—small, cost-effective adjustments over time—can help maintain system engagement without imposing high upfront costs. This finding underscores the importance of cost management in TIP design, contributing valuable guidance for manufacturers and policymakers on how to allocate resources for maximum sustainability impact.

The parameter *ψ*_1_, representing brand reputation, social responsibility, and government subsidies, significantly accelerates the system’s progression to a stable equilibrium (GI, PS, CP). This finding supports the hypothesis regarding external influences, showing that brand reputation and government support can play essential roles in fostering TIP participation. Real-world applications include publicizing the environmental benefits of TIPs, which can attract customer participation and strengthen the manufacturer’s reputation as a socially responsible entity. Government subsidies further amplify this effect by adding financial incentives that support TIP growth. Manufacturers should actively promote their brand’s environmental commitment by engaging in TIPs, using media campaigns to highlight TIP benefits. Policymakers can further bolster this impact by offering recognition or rewards to companies with strong TIP engagement, fostering industry-wide support for sustainable practices. This conclusion highlights how brand reputation and government involvement can amplify TIP effectiveness, offering strategic insights for manufacturers to improve stakeholder cooperation and reinforce sustainable practices.

In conclusion, this study emphasizes the dominant role manufacturers play in TIP success through effective use of incentives, revenue-sharing, and innovation strategies. The EGM framework offers a dynamic model for understanding TIP interactions, allowing industry leaders to make data-informed decisions that enhance TIP sustainability. Additionally, by examining the decision-making processes of recyclers and customers, this study provides valuable insights that bridge theoretical understanding with practical application, reinforcing TIP design as a fundamental component of sustainable recycling in logistics and supply chain management. Policymakers should support TIPs by crafting policies that encourage manufacturer-led incentives and establish standards for sustainable recycling practices. Industry leaders can use these findings to refine TIP strategies, creating circular economy frameworks that drive sustainable development. These practical implications and recommendations guide industry practitioners and policymakers in designing TIPs that promote recycling, support stakeholder engagement, and advance sustainability in logistics and the circular economy.

## Supporting information

S1 File (ZIP)
