# Supplementary material for: Behavioral choice of manufacturers, recyclers and customers in Trade-In Programs
Source: PLoS One. 2024 Dec 30;19(12):e0316344. doi: 10.1371/journal.pone.0316344 (PMC11684666; doi:10.1371/journal.pone.0316344)
Supplement: S1 File — (ZIP) [file pone.0316344.s001.zip › Programs/Calculation program-Jacobi matrix of the dynamical system-Equilibrium points.docx]

1. **Find the dynamic equation of replication**

clc; clear;

syms x y z a b d r E R F1 F2 F3 S1 S2 C1 C2 T

% **Calculate F(x)**

E11=y*z*(F1+b*F2+a*R+r*E-C1-S1-S2-T)+y*(1-z)*(F1+F3+a*R-C1-S1-T)+(1-y)*z*(F1+F2+R-C1-d-S2-T)+(1-y)*(1-z)*(F1+F3+a*R-C1-T);%¼Ó´Ö±äÁ¿×ÔÐÐ¸ü¸Ä

E12=y*z*(b*F2+a*R-C2)+y*(1-z)*(F3+a*R-C2)+(1-y)*z*(F2+R-d-C2)+(1-y)*(1-z)*(F3+a*R-C2);%¼Ó´Ö±äÁ¿×ÔÐÐ¸ü¸Ä

E1=simplify(x*E11+(1-x)*E12)%ÇóÆ½¾ùÆÚÍû

F(x)=simplify(x*(E11-E1))%Çó¸´ÖÆ¶¯Ì¬·½³Ì

%F(x) =x*(x - 1)*(C1 - C2 - F1 + T + S1*y + S2*z - E*r*y*z)

%%%%%%%%%%%%%%%%%%%%%%%%%%%%%%%%%%

% **Calculate F(y)**

clc;clear;

syms x y z a b d r E R F1 F2 F3 S1 S2 C1 C2 C3

E21=x*z*((1-b)*F2+(1-a)*R+(1-r)*E+S1-C3)+x*(1-z)*((1-a)*R+S1-C3)+(1-x)*z*((1-b)*F2+(1-a)*R-C3)+(1-x)*(1-z)*((1-a)*R-C3);%¼Ó´Ö±äÁ¿×ÔÐÐ¸ü¸Ä

E22=(1-x)*(1-z)*(1-a)*R+x*(1-z)*(1-a)*R;%¼Ó´Ö±äÁ¿×ÔÐÐ¸ü¸Ä

E2=simplify(y*E21+(1-y)*E22)%ÇóÆ½¾ùÆÚÍû

F(y)=simplify(y*(E21-E2))%Çó¸´ÖÆ¶¯Ì¬·½³Ì

%F(y) =y*(y - 1)*(C3 - F2*z - S1*x - R*z + F2*b*z + R*a*z - E*x*z + E*r*x*z)

%%%%%%%%%%%%%%%%%%%%%%%%%%%%%%%%%%

% **Calculate F(z)**

clc;clear;

syms x y z a b d K R P F1 F2 F3 S1 S2 C1 C2 C3 C4

E31=x*y*(K-C4+S2)+x*(1-y)*(K-C4+S2)+(1-x)*y*(K-C4)+(1-x)*(1-y)*(K-C4);%¼Ó´Ö±äÁ¿×ÔÐÐ¸ü¸Ä

E32=x*y*P+x*(1-y)*P+(1-x)*y*P+(1-x)*(1-y)*P;%¼Ó´Ö±äÁ¿×ÔÐÐ¸ü¸Ä

E3=simplify(z*E31+(1-z)*E32)%ÇóÆ½¾ùÆÚÍû

F(z)=simplify(z*(E31-E3))%Çó¸´ÖÆ¶¯Ì¬·½³Ì

%F(z) =z*(z - 1)*(C4 - K + P - S2*x)

%%%%%%%%%%%%%%%%%%%%%%%%%%%%%%%%%%%%%%%

% **Calculate the derivative of F(x)**

clc;clear;

syms x y z a b d r E R F1 F2 F3 S1 S2 C1 C2 T;

f=x*(1-x)*(C1-C2-F1+T+S1*y+S2*z-E*r*y*z);

dfx=simplify(diff(f,x))

dfy=simplify(diff(f, y))

dfz=simplify(diff(f, z))

%dfx =-(2*x - 1)*(C1 - C2 - F1 + T + S1*y + S2*z - E*r*y*z)

%dfy =-x*(S1 - E*r*z)*(x - 1)

%dfz =-x*(S2 - E*r*y)*(x - 1)

%%%%%%%%%%%%%%%% **Calculate the derivative of F(y)**

clc;clear;

syms x y z a b d r E R F1 F2 F3 S1 S2 C1 C2 C3 T;

f=y*(y-1)*(C3-F2*z-S1*x-R*z+F2*b*z+R*a*z-E*x*z+E*r*x*z);

dfx=simplify(diff(f,x))

dfy=simplify(diff(f, y))

dfz=simplify(diff(f, z))

%dfx =-y*(y - 1)*(S1 + E*z - E*r*z)

%dfy =(2*y - 1)*(C3 - F2*z - S1*x - R*z + F2*b*z + R*a*z - E*x*z + E*r*x*z)

%dfz =-y*(y - 1)*(F2 + R - F2*b - R*a + E*x - E*r*x)

%%%%%%%%%%%%%%%% **Calculate the derivative of F(Z)**

clc;clear;

syms x y z a b d K R P F1 F2 F3 S1 S2 C1 C2 C3 C4

f=z*(z-1)*(C4-K+P-S2*x);

dfx=simplify(diff(f,x))

dfy=simplify(diff(f, y))

dfz=simplify(diff(f, z))

%dfx =-S2*z*(z - 1)

%dfy =0

%dfz =(2*z - 1)*(C4 - K + P - S2*x)

%%%%%%%%%%%%%%%%%%%%%%%%%%%%%%%%%%%%%%%%

%**- Solve the Jacobian matrix eigenvalues**

% **Equilibrium point E1**

clc;clear;

syms x y z a b d r K R P F1 F2 F3 S1 S2 C1 C2 C3 C4 T E;

x=0,y=0,z=0;

A = [(2*x-1)*(C1-C2-F1+T+S1*y+S2*z-E*r*y*z) x*(x-1)*(E*r*z-S1) x*(x-1)*(E*r*y-S2);

y*(y-1)*( E*r*z-S1-E*z) (2*y-1)*(C3-F2*z-S1*x-R*z+F2*b*z+R*a*z-E*x*z+E*r*x*z) y*(y-1)*(F2*b+R*a+E*r*x-F2-R-E*x);

z*(z-1)*(-S2) 0 (2*z-1)*(C4-K+P-S2*x)];

[V,R] = eig(A),DA=det(A),IA=inv(A),

% **Equilibrium point E2**

clc;clear;

syms x y z a b d r K R P F1 F2 F3 S1 S2 C1 C2 C3 C4 T E;

x=1,y=0,z=0; %¾ùºâµã

A = [(2*x-1)*(C1-C2-F1+T+S1*y+S2*z-E*r*y*z) x*(x-1)*(E*r*z-S1) x*(x-1)*(E*r*y-S2);

y*(y-1)*( E*r*z-S1-E*z) (2*y-1)*(C3-F2*z-S1*x-R*z+F2*b*z+R*a*z-E*x*z+E*r*x*z) y*(y-1)*(F2*b+R*a+E*r*x-F2-R-E*x);

z*(z-1)*(-S2) 0 (2*z-1)*(C4-K+P-S2*x)];

[V,R] = eig(A),DA=det(A),IA=inv(A),

% **Equilibrium point E3**

clc;clear;

syms x y z a b d r K R P F1 F2 F3 S1 S2 C1 C2 C3 C4 T E;

x=0,y=1,z=0; %¾ùºâµã

A = [(2*x-1)*(C1-C2-F1+T+S1*y+S2*z-E*r*y*z) x*(x-1)*(E*r*z-S1) x*(x-1)*(E*r*y-S2);

y*(y-1)*( E*r*z-S1-E*z) (2*y-1)*(C3-F2*z-S1*x-R*z+F2*b*z+R*a*z-E*x*z+E*r*x*z) y*(y-1)*(F2*b+R*a+E*r*x-F2-R-E*x);

z*(z-1)*(-S2) 0 (2*z-1)*(C4-K+P-S2*x)];

[V,R] = eig(A),DA=det(A),IA=inv(A),

% **Equilibrium point E4**

clc;clear;

syms x y z a b d r K R P F1 F2 F3 S1 S2 C1 C2 C3 C4 T E;

x=0,y=0,z=1; %¾ùºâµã

A = [(2*x-1)*(C1-C2-F1+T+S1*y+S2*z-E*r*y*z) x*(x-1)*(E*r*z-S1) x*(x-1)*(E*r*y-S2);

y*(y-1)*( E*r*z-S1-E*z) (2*y-1)*(C3-F2*z-S1*x-R*z+F2*b*z+R*a*z-E*x*z+E*r*x*z) y*(y-1)*(F2*b+R*a+E*r*x-F2-R-E*x);

z*(z-1)*(-S2) 0 (2*z-1)*(C4-K+P-S2*x)];

[V,R] = eig(A),DA=det(A),IA=inv(A),

% **Equilibrium point E5**

clc;clear;

syms x y z a b d r K R P F1 F2 F3 S1 S2 C1 C2 C3 C4 T E;

x=1,y=1,z=0; %¾ùºâµã

A = [(2*x-1)*(C1-C2-F1+T+S1*y+S2*z-E*r*y*z) x*(x-1)*(E*r*z-S1) x*(x-1)*(E*r*y-S2);

y*(y-1)*( E*r*z-S1-E*z) (2*y-1)*(C3-F2*z-S1*x-R*z+F2*b*z+R*a*z-E*x*z+E*r*x*z) y*(y-1)*(F2*b+R*a+E*r*x-F2-R-E*x);

z*(z-1)*(-S2) 0 (2*z-1)*(C4-K+P-S2*x)];

[V,R] = eig(A),DA=det(A),IA=inv(A),

% **Equilibrium point E6**

clc;clear;

syms x y z a b d r K R P F1 F2 F3 S1 S2 C1 C2 C3 C4 T E;

x=1,y=0,z=1; %¾ùºâµã

A = [(2*x-1)*(C1-C2-F1+T+S1*y+S2*z-E*r*y*z) x*(x-1)*(E*r*z-S1) x*(x-1)*(E*r*y-S2);

y*(y-1)*( E*r*z-S1-E*z) (2*y-1)*(C3-F2*z-S1*x-R*z+F2*b*z+R*a*z-E*x*z+E*r*x*z) y*(y-1)*(F2*b+R*a+E*r*x-F2-R-E*x);

z*(z-1)*(-S2) 0 (2*z-1)*(C4-K+P-S2*x)];

[V,R] = eig(A),DA=det(A),IA=inv(A),

% **Equilibrium point E7**

clc;clear;

syms x y z a b d r K R P F1 F2 F3 S1 S2 C1 C2 C3 C4 T E;

x=0,y=1,z=1; %¾ùºâµã

A = [(2*x-1)*(C1-C2-F1+T+S1*y+S2*z-E*r*y*z) x*(x-1)*(E*r*z-S1) x*(x-1)*(E*r*y-S2);

y*(y-1)*( E*r*z-S1-E*z) (2*y-1)*(C3-F2*z-S1*x-R*z+F2*b*z+R*a*z-E*x*z+E*r*x*z) y*(y-1)*(F2*b+R*a+E*r*x-F2-R-E*x);

z*(z-1)*(-S2) 0 (2*z-1)*(C4-K+P-S2*x)];

[V,R] = eig(A),DA=det(A),IA=inv(A),

% **Equilibrium point E8**

clc;clear;

syms x y z a b d r K R P F1 F2 F3 S1 S2 C1 C2 C3 C4 T E;

x=1,y=1,z=1; %¾ùºâµã

A = [(2*x-1)*(C1-C2-F1+T+S1*y+S2*z-E*r*y*z) x*(x-1)*(E*r*z-S1) x*(x-1)*(E*r*y-S2);

y*(y-1)*( E*r*z-S1-E*z) (2*y-1)*(C3-F2*z-S1*x-R*z+F2*b*z+R*a*z-E*x*z+E*r*x*z) y*(y-1)*(F2*b+R*a+E*r*x-F2-R-E*x);

z*(z-1)*(-S2) 0 (2*z-1)*(C4-K+P-S2*x)];

[V,R] = eig(A),DA=det(A),IA=inv(A),
