# Supplementary material for: Behavioral choice of manufacturers, recyclers and customers in Trade-In Programs
Source: PLoS One. 2024 Dec 30;19(12):e0316344. doi: 10.1371/journal.pone.0316344 (PMC11684666; doi:10.1371/journal.pone.0316344)
Supplement: S1 File — (ZIP) [file pone.0316344.s001.zip › Programs/Fig2-Fig3.docx]

%%%%%%%%%%%% **Fig 2. Dynamics trend of manufacturers**

clc,clear;

figure(1)

[x,y]=meshgrid(0:0.001:1,0:0.001:1);

%W1=20,R11=60,R12=58;

T=80,F1=150,S1=20,S2=15,C1=15,C2=10;

z=-(C2+F1-C1-T-S1*y)./(S2)

mesh(x,y,z)

colormap(cool)

alpha(0.0001)

view([100 40]);

set(gca,'XTick',[0:0.2:1],'YTick',[0:0.3:1],'ZTick',[0:0.4:1])

axis([0 1 0 1 0 1])

xlabel('$x$','interpreter','latex');

ylabel('$y$','interpreter','latex');

zlabel('$z$','interpreter','latex','Rotation',360);

grid on

hold on

set(0,'defaultfigurecolor','w')

text(0.5 ,0.2 ,0.3,'$(a)y=y^{*}$','interpreter','latex');

text(0.4 ,0.2 ,0.3,'$(b)y<y^{*},x\rightarrow0$','interpreter','latex');

text(0.3 ,0.2 ,0.3,'$(c)y>y^{*},x\rightarrow1$','interpreter','latex');

annotation('arrow',[0.55 0.35],[0.35 0.32]);

annotation('arrow',[0.58 0.58],[0.38 0.45]);

%%%%%%%%%%%% **Fig 3. Dynamics trend of the recyclers**

clc,clear;

figure(2)

[x,y]=meshgrid(0:0.01:1,0:0.01:1);

%C2=10,W1=20,W2=15,W3=25;

a=0.7,b=0.8,R=150,F2=350,S1=20,S2=15,C3=55;

%z=-(C3-S1*x)./(F2+R-F2*b-a*R);ÕæÊµÖµ

z=(C3-S1*x)./(F2+R-F2*b-a*R); %ÃÀ»¯Í¼

mesh(x,y,z)

colormap(pink)

alpha(0.0001)

%surf(x,y,z)

view([100 40]);

set(gca,'XTick',[0:0.5:1],'YTick',[0:0.2:1],'ZTick',[0:0.1:1])

axis([0 1 0 1 0 1])

xlabel('$x$','interpreter','latex');

ylabel('$y$','interpreter','latex');

zlabel('$z$','interpreter','latex','Rotation',360);

grid on

hold on

set(0,'defaultfigurecolor','w')

text(0.5 ,0 ,0.8,'$(a)z=z^{*}$','interpreter','latex');

text(0.4 ,0 ,0.8,'$(b)z<z^{*},y\rightarrow0$','interpreter','latex');

text(0.8 ,0, 0.8,'$(c)z>z^{*},y\rightarrow1$','interpreter','latex');

annotation('arrow',[0.55 0.35],[0.35 0.32]);

annotation('arrow',[0.58 0.58],[0.38 0.45]);

%title('Í¼2 ÏàÎ»Í¼');
