# Supplementary material for: Behavioral choice of manufacturers, recyclers and customers in Trade-In Programs
Source: PLoS One. 2024 Dec 30;19(12):e0316344. doi: 10.1371/journal.pone.0316344 (PMC11684666; doi:10.1371/journal.pone.0316344)
Supplement: S1 File — (ZIP) [file pone.0316344.s001.zip › Programs/Fig4-Fig6.docx]

1. **Fig 4. Effect of Initial Setting.**

clc,clear;

figure(1);

a=0.7,b=0.8,K=100,R=150,P=20,T=80,F1=150,F2=350,S1=20,S2=15,C1=25,C2=10,C3=55,C4=5;

[t,y]=ode45(@(t,y) manufacturer1(t,y,a,b,K,R,P,T,F1,F2,S1,S2,C1,C2,C3,C4),[0 1],[0.5 0.5 0.5]);

points=1:2:length(t);

plot(y(:,1),'rh-','linewidth',1,'markersize',5,'markerfacecolor','r','markerindices',points);

hold on;

[t,y]=ode45(@(t,y) manufacturer1(t,y,a,b,K,R,P,T,F1,F2,S1,S2,C1,C2,C3,C4),[0 1],[0.5 0.5 0.5]);

points=1:2:length(t);

plot(y(:,2),'g--','linewidth',1,'markersize',5,'markerindices',points);

hold on;

[t,y]=ode45(@(t,y) manufacturer1(t,y,a,b,K,R,P,T,F1,F2,S1,S2,C1,C2,C3,C4),[0 1],[0.5 0.5 0.5]);

points=1:1:length(t);

plot(y(:,3),'b:','linewidth',1,'markersize',5,'markerindices',points);

hold on;

set(0,'defaultfigurecolor','w')

grid on

hold on

xlabel('$Time$','interpreter','latex','Rotation',0.5);

ylabel('$Proportion$','interpreter','latex');

legend('x','y','z');

**%%%%%%%%%%%%%%%%%%%%%%%%%%%%%%%%%%%%%%%%%%%%%%%%%%%%%%**

1. **Fig 5. The impact of initial probability of manufacturers on recyclers and consumers**

clc,clear;

figure(1);

subplot(2,2,1);

a=0.7,b=0.8,K=100,R=150,P=20,T=80,F1=150,F2=350,S1=20,S2=15,C1=25,C2=10,C3=55,C4=5;

[t,y]=ode45(@(t,y) manufacturer1(t,y,a,b,K,R,P,T,F1,F2,S1,S2,C1,C2,C3,C4),[0 1],[0.1 0.5 0.5]);

points=1:2:length(t); %ÖÐ¼äµÄ1¸ÄÎª´óÒ»µãµÄÕýÕûÊý£¬ÄÇÃ´Í¼ÏóÉÏµÄµã¾Í»á±äµÃÏ¡Êè

plot(t,y(:,2),'rh-','linewidth',1,'markersize',5,'markerindices',points);

hold on;

[t,y]=ode45(@(t,y) manufacturer1(t,y,a,b,K,R,P,T,F1,F2,S1,S2,C1,C2,C3,C4),[0 1],[0.5 0.5 0.5]);

points=1:2:length(t);

plot(t,y(:,2),'b:','linewidth',1,'markersize',5,'markerindices',points);

hold on;

[t,y]=ode45(@(t,y) manufacturer1(t,y,a,b,K,R,P,T,F1,F2,S1,S2,C1,C2,C3,C4),[0 1],[0.9 0.5 0.5]);

points=1:2:length(t);

plot(t,y(:,2),'m--','linewidth',1,'markersize',5,'markerindices',points);

hold on;

set(0,'defaultfigurecolor','w')

grid on

hold on

xlabel('$t$','interpreter','latex','Rotation',0);

ylabel('$y$','interpreter','latex');

set(gca,'XTick',[0:0.2:1],'YTick',[0:0.2:1])

axis([0 1 0 1]);

legend('x=0.1','x=0.5','x=0.9');

subplot(2,2,2);

a=0.7,b=0.8,K=100,R=150,P=20,T=80,F1=150,F2=350,S1=20,S2=15,C1=25,C2=10,C3=55,C4=5;

[t,y]=ode45(@(t,y) manufacturer1(t,y,a,b,K,R,P,T,F1,F2,S1,S2,C1,C2,C3,C4),[0 1],[0.1 0.5 0.5]);

points=1:2:length(t); %ÖÐ¼äµÄ1¸ÄÎª´óÒ»µãµÄÕýÕûÊý£¬ÄÇÃ´Í¼ÏóÉÏµÄµã¾Í»á±äµÃÏ¡Êè

plot(t,y(:,3),'rh:','linewidth',1,'markersize',5,'markerindices',points);

hold on;

[t,y]=ode45(@(t,y) manufacturer1(t,y,a,b,K,R,P,T,F1,F2,S1,S2,C1,C2,C3,C4),[0 1],[0.5 0.5 0.5]);

points=1:2:length(t);

plot(t,y(:,3),'b:','linewidth',1,'markersize',5,'markerindices',points);

hold on;

[t,y]=ode45(@(t,y) manufacturer1(t,y,a,b,K,R,P,T,F1,F2,S1,S2,C1,C2,C3,C4),[0 1],[0.9 0.5 0.5]);

points=1:2:length(t);

plot(t,y(:,3),'m--','linewidth',1,'markersize',5,'markerindices',points);

hold on;

%°×µ×£¬Íø¸ñ

set(0,'defaultfigurecolor','w')

grid on

hold on

%×ø±ê±ê×¢£¬¿Ì¶È¼ä¸ô¼°Çø¼ä

xlabel('$t$','interpreter','latex','Rotation',0);

ylabel('$z$','interpreter','latex');

set(gca,'XTick',[0:0.05:1],'YTick',[0:0.05:1])

axis([0 1 0 1]);

%Í¼Àý£¬±êÌâ

legend('x=0.1','x=0.5','x=0.9');

title('Fig.6.Effect of initial probability of manufacturers on recyclers','position',[-0.1 -0.3]','FontWeight','bold');

%%%%%%%%%%%%%%%%%%%%%%%%%%%%%%%%%%%%%%%%%%%%%%%%%%%%%%

1ÒÔÍ¼5ÎªÀý£¬Ò»ÖÖÊÇ¸Ä±äx,y,zµÄ³õÊ¼Öµ£¬³õÊ¼Öµ³£¼ûÈ¡ÖµÎª0.5,0.2,0.1,0.9£¬ÕâÀïÎÒÃÇÒÔ0.2,0.5,0.7ÎªÀý

%%Æ½Ãæy-t yµÄÓ°Ïì

clc,clear;

figure(1);

subplot(2,2,1);

%ÏßÌõ

a=0.7,b=0.8,K=100,R=150,P=20,T=80,F1=150,F2=350,S1=20,S2=15,C1=25,C2=10,C3=55,C4=5;

[t,y]=ode45(@(t,y) manufacturer1(t,y,a,b,K,R,P,T,F1,F2,S1,S2,C1,C2,C3,C4),[0 1],[0.5 0.1 0.5]);

points=1:2:length(t); %ÖÐ¼äµÄ1¸ÄÎª´óÒ»µãµÄÕýÕûÊý£¬ÄÇÃ´Í¼ÏóÉÏµÄµã¾Í»á±äµÃÏ¡Êè

plot(t,y(:,1),'rh-','linewidth',1,'markersize',5,'markerindices',points);

hold on;

[t,y]=ode45(@(t,y) manufacturer1(t,y,a,b,K,R,P,T,F1,F2,S1,S2,C1,C2,C3,C4),[0 1],[0.5 0.5 0.5]);

points=1:2:length(t);

plot(t,y(:,1),'b:','linewidth',1,'markersize',5,'markerindices',points);

hold on;

[t,y]=ode45(@(t,y) manufacturer1(t,y,a,b,K,R,P,T,F1,F2,S1,S2,C1,C2,C3,C4),[0 1],[0.5 0.9 0.5]);

points=1:2:length(t);

plot(t,y(:,1),'m--','linewidth',1,'markersize',5,'markerindices',points);

hold on;

%°×µ×£¬Íø¸ñ

set(0,'defaultfigurecolor','w')

grid on

hold on

%×ø±ê±ê×¢£¬¿Ì¶È¼ä¸ô¼°Çø¼ä

xlabel('$t$','interpreter','latex','Rotation',0);

ylabel('$x$','interpreter','latex');

set(gca,'XTick',[0:0.2:1],'YTick',[0:0.2:1])

axis([0 1 0 1]);

legend('y=0.1','y=0.5','y=0.9');

subplot(2,2,2);

%ÏßÌõ

a=0.7,b=0.8,K=100,R=150,P=20,T=80,F1=150,F2=350,S1=20,S2=15,C1=25,C2=10,C3=55,C4=5;

[t,y]=ode45(@(t,y) manufacturer1(t,y,a,b,K,R,P,T,F1,F2,S1,S2,C1,C2,C3,C4),[0 1],[0.5 0.1 0.5]);

points=1:2:length(t); %ÖÐ¼äµÄ1¸ÄÎª´óÒ»µãµÄÕýÕûÊý£¬ÄÇÃ´Í¼ÏóÉÏµÄµã¾Í»á±äµÃÏ¡Êè

plot(t,y(:,3),'rh:','linewidth',1,'markersize',5,'markerindices',points);

hold on;

[t,y]=ode45(@(t,y) manufacturer1(t,y,a,b,K,R,P,T,F1,F2,S1,S2,C1,C2,C3,C4),[0 1],[0.5 0.5 0.5]);

points=1:2:length(t);

plot(t,y(:,3),'b:','linewidth',1,'markersize',5,'markerindices',points);

hold on;

[t,y]=ode45(@(t,y) manufacturer1(t,y,a,b,K,R,P,T,F1,F2,S1,S2,C1,C2,C3,C4),[0 1],[0.5 0.9 0.5]);

points=1:2:length(t);

plot(t,y(:,3),'m--','linewidth',1,'markersize',5,'markerindices',points);

hold on;

%°×µ×£¬Íø¸ñ

set(0,'defaultfigurecolor','w')

grid on

hold on

%×ø±ê±ê×¢£¬¿Ì¶È¼ä¸ô¼°Çø¼ä

xlabel('$t$','interpreter','latex','Rotation',0);

ylabel('$z$','interpreter','latex');

set(gca,'XTick',[0:0.05:1],'YTick',[0:0.05:1])

axis([0 1 0 1]);

%Í¼Àý£¬±êÌâ

legend('y=0.1','y=0.5','y=0.9');

%title('Fig.6.Effect of initial probability of manufacturers on recyclers','position',[-0.1 -0.3]','FontWeight','bold');

%%%%%%%%%%%%%%%%%%%%%%%%%%%%%%%%%%%%%%%%%%%%%%%%%%%%%%

1. **Fig 6. The impact of initial probability of recyclers on manufacturers and consumers**

clc,clear;

figure(1);

subplot(2,2,1);

%ÏßÌõ

a=0.7,b=0.8,K=100,R=150,P=20,T=80,F1=150,F2=350,S1=20,S2=15,C1=25,C2=10,C3=55,C4=5;

[t,y]=ode45(@(t,y) manufacturer1(t,y,a,b,K,R,P,T,F1,F2,S1,S2,C1,C2,C3,C4),[0 1],[0.5 0.5 0.1]);

points=1:2:length(t); %ÖÐ¼äµÄ1¸ÄÎª´óÒ»µãµÄÕýÕûÊý£¬ÄÇÃ´Í¼ÏóÉÏµÄµã¾Í»á±äµÃÏ¡Êè

plot(t,y(:,1),'rh-','linewidth',1,'markersize',5,'markerindices',points);

hold on;

[t,y]=ode45(@(t,y) manufacturer1(t,y,a,b,K,R,P,T,F1,F2,S1,S2,C1,C2,C3,C4),[0 1],[0.5 0.5 0.5]);

points=1:2:length(t);

plot(t,y(:,1),'b:','linewidth',1,'markersize',5,'markerindices',points);

hold on;

[t,y]=ode45(@(t,y) manufacturer1(t,y,a,b,K,R,P,T,F1,F2,S1,S2,C1,C2,C3,C4),[0 1],[0.5 0.5 0.9]);

points=1:2:length(t);

plot(t,y(:,1),'m--','linewidth',1,'markersize',5,'markerindices',points);

hold on;

%°×µ×£¬Íø¸ñ

set(0,'defaultfigurecolor','w')

grid on

hold on

%×ø±ê±ê×¢£¬¿Ì¶È¼ä¸ô¼°Çø¼ä

xlabel('$t$','interpreter','latex','Rotation',0);

ylabel('$x$','interpreter','latex');

set(gca,'XTick',[0:0.2:1],'YTick',[0:0.2:1])

axis([0 1 0 1]);

legend('z=0.1','z=0.5','z=0.9');

subplot(2,2,2);

%ÏßÌõ

a=0.7,b=0.8,K=100,R=150,P=20,T=80,F1=150,F2=350,S1=20,S2=15,C1=25,C2=10,C3=55,C4=5;

[t,y]=ode45(@(t,y) manufacturer1(t,y,a,b,K,R,P,T,F1,F2,S1,S2,C1,C2,C3,C4),[0 1],[0.5 0.5 0.1]);

points=1:2:length(t); %ÖÐ¼äµÄ1¸ÄÎª´óÒ»µãµÄÕýÕûÊý£¬ÄÇÃ´Í¼ÏóÉÏµÄµã¾Í»á±äµÃÏ¡Êè

plot(t,y(:,2),'rh:','linewidth',1,'markersize',5,'markerindices',points);

hold on;

[t,y]=ode45(@(t,y) manufacturer1(t,y,a,b,K,R,P,T,F1,F2,S1,S2,C1,C2,C3,C4),[0 1],[0.5 0.5 0.5]);

points=1:2:length(t);

plot(t,y(:,2),'b:','linewidth',1,'markersize',5,'markerindices',points);

hold on;

[t,y]=ode45(@(t,y) manufacturer1(t,y,a,b,K,R,P,T,F1,F2,S1,S2,C1,C2,C3,C4),[0 1],[0.5 0.5 0.9]);

points=1:2:length(t);

plot(t,y(:,2),'m--','linewidth',1,'markersize',5,'markerindices',points);

hold on;

%°×µ×£¬Íø¸ñ

set(0,'defaultfigurecolor','w')

grid on

hold on

%×ø±ê±ê×¢£¬¿Ì¶È¼ä¸ô¼°Çø¼ä

xlabel('$t$','interpreter','latex','Rotation',0);

ylabel('$y$','interpreter','latex');

set(gca,'XTick',[0:0.05:1],'YTick',[0:0.05:1])

axis([0 1 0 1]);

%Í¼Àý£¬±êÌâ

legend('z=0.1','z=0.5','z=0.9');

%title('Fig.6.Effect of initial probability of manufacturers on recyclers','position',[-0.1 -0.3]','FontWeight','bold');
