# Supplementary material for: Behavioral choice of manufacturers, recyclers and customers in Trade-In Programs
Source: PLoS One. 2024 Dec 30;19(12):e0316344. doi: 10.1371/journal.pone.0316344 (PMC11684666; doi:10.1371/journal.pone.0316344)
Supplement: S1 File — (ZIP) [file pone.0316344.s001.zip › Programs/Fig7-Fig15.docx]

1. **Fig 8 The influence of manufacturers’ incentive costs** $\boldsymbol{C}_{\boldsymbol{1}}$

clc,clear;

figure(8);

%C1=15

a=0.7,b=0.8,K=100,R=150,P=20,T=80,F1=150,F2=350,S1=20,S2=15,C1=15,C2=10,C3=55,C4=5;

[t,y]=ode45(@(t,y) manufacturer1(t,y,a,b,K,R,P,T,F1,F2,S1,S2,C1,C2,C3,C4),[0 50],[0.2 0.2 0.2]);

plot3(y(:,1),y(:,2),y(:,3),'g--','linewidth',0.5);

hold on ;

%C1=25

a=0.7,b=0.8,K=100,R=150,P=20,T=80,F1=150,F2=350,S1=20,S2=15,C1=25,C2=10,C3=55,C4=5;

[t,y]=ode45(@(t,y) manufacturer1(t,y,a,b,K,R,P,T,F1,F2,S1,S2,C1,C2,C3,C4),[0 50],[0.2 0.2 0.2]);

plot3(y(:,1),y(:,2),y(:,3),'r+','linewidth',1);

hold on ;

%C1=40

a=0.7,b=0.8,K=100,R=150,P=20,T=80,F1=150,F2=350,S1=20,S2=15,C1=40,C2=10,C3=55,C4=5;

[t,y]=ode45(@(t,y) manufacturer1(t,y,a,b,K,R,P,T,F1,F2,S1,S2,C1,C2,C3,C4),[0 50],[0.2 0.2 0.2]);

plot3(y(:,1),y(:,2),y(:,3),'b-','linewidth',1);

hold on ;

set(gca,'XTick',[0:0.2:1],'YTick',[0:0.2:1],'ZTick',[0:0.2:1])

axis([0 1 0 1 0 1])

xlabel('$x$','interpreter','latex');ylabel('$y$','interpreter','latex');zlabel('$z$','interpreter','latex','Rotation',360,'position',[-0.1 1 1.1]);

grid on

hold on

set(0,'defaultfigurecolor','w')

legend({'{\it\fontname{Bodoni MT}C_{1}}=15','{\it\fontname{Bodoni MT}C_{1}}=25','{\it\fontname{Bodoni MT}C_{1}}=40'},'location','northeast');

title('Fig. 8. The influence of the incentives cost C_{1}. ','FontWeight','bold','position',[0 0 -0.2]);

text(0.4 ,0.2 ,0.3,'$ESS$','interpreter','latex');

annotation('arrow',[0.55 0.35],[0.35 0.32]);

annotation('arrow',[0.58 0.58],[0.38 0.45]);

% the small figure

axes('position',[0.13 0.32 0.2 0.2]); %Ð¡Í¼µÄ¹Ø¼üÓï¾äÈ·¶¨Ð¡Í¼µÄ´óÐ¡Î»ÖÃ

% z-xÐ¡Í¼ÖÐµÄÏßÌõ

a=0.7,b=0.8,K=100,R=150,P=20,T=80,F1=150,F2=350,S1=20,S2=15,C1=15,C2=10,C3=55,C4=5;

[t,y]=ode45(@(t,y) manufacturer1(t,y,a,b,K,R,P,T,F1,F2,S1,S2,C1,C2,C3,C4),[0 50],[0.2 0.2 0.2]);

plot3(y(:,1),y(:,2),y(:,3),'r+','linewidth',1);

hold on

a=0.7,b=0.8,K=100,R=150,P=20,T=80,F1=150,F2=350,S1=20,S2=15,C1=25,C2=10,C3=55,C4=5;

[t,y]=ode45(@(t,y) manufacturer1(t,y,a,b,K,R,P,T,F1,F2,S1,S2,C1,C2,C3,C4),[0 50],[0.2 0.2 0.2]);

plot3(y(:,1),y(:,2),y(:,3),'b-','linewidth',1);

% stem3(y(:,1),y(:,2),y(:,3),'g--','linewidth',1);%»ð²ñ¹÷Í¼

hold on

a=0.7,b=0.8,K=100,R=150,P=20,T=80,F1=150,F2=350,S1=20,S2=15,C1=40,C2=10,C3=55,C4=5;

[t,y]=ode45(@(t,y) manufacturer1(t,y,a,b,K,R,P,T,F1,F2,S1,S2,C1,C2,C3,C4),[0 50],[0.2 0.2 0.2]);

plot3(y(:,1),y(:,2),y(:,3),'g--','linewidth',1);

% stem3(y(:,1),y(:,2),y(:,3),'g--','linewidth',1);%»ð²ñ¹÷Í¼

hold on

%×ø±ê¿Ì¶È¼ä¸ô¼°Çø¼ä£¬µ«²»ÏÔÊ¾

set(gca,'XTick',[0:0.2:1],'YTick',[0:0.2:1],'ZTick',[0:0.2:1])

axis([0 1 0 1 0 1])

set(gca,'XTickLabel','','YTickLabel','','ZTickLabel','')

%±³¾°Íø¸ñ²¢ÉèÖÃ°×µ×

grid on

hold on

set(0,'defaultfigurecolor','w')

%Í¼ÏóÏÔÊ¾ÊÓ½Ç£¬ÒÔ¼°ÏàÓ¦µÄ×ø±ê±ê×¢

view([0 0]); %z-xÐ¡Í¼

xlabel('x','position',[0.8 1 0.3])

zlabel('z','position',[0.1 1 0.8],'Rotation',360)

1. **Fig 9. The influence of revenue-sharing coefficients** $\boldsymbol{\alpha}$

clc,clear;

figure(9);

%a=0.5

a=0.5,b=0.8,K=100,R=150,P=20,T=80,F1=150,F2=350,S1=20,S2=15,C1=25,C2=10,C3=55,C4=5;

[t,y]=ode45(@(t,y) manufacturer1(t,y,a,b,K,R,P,T,F1,F2,S1,S2,C1,C2,C3,C4),[0 50],[0.2 0.2 0.2]);

plot3(y(:,1),y(:,2),y(:,3),'r+','linewidth',1);

hold on ;

%a=0.7

a=0.7,b=0.8,K=100,R=150,P=20,T=80,F1=150,F2=350,S1=20,S2=15,C1=25,C2=10,C3=55,C4=5;

[t,y]=ode45(@(t,y) manufacturer1(t,y,a,b,K,R,P,T,F1,F2,S1,S2,C1,C2,C3,C4),[0 50],[0.2 0.2 0.2]);

plot3(y(:,1),y(:,2),y(:,3),'b-','linewidth',1);

hold on ;

%a=0.8

a=0.8,b=0.8,K=100,R=150,P=20,T=80,F1=150,F2=350,S1=20,S2=15,C1=25,C2=10,C3=55,C4=5;

[t,y]=ode45(@(t,y) manufacturer1(t,y,a,b,K,R,P,T,F1,F2,S1,S2,C1,C2,C3,C4),[0 50],[0.2 0.2 0.2]);

plot3(y(:,1),y(:,2),y(:,3),'g--','linewidth',1);

hold on ;

%×ø±ê¿Ì¶È¼ä¸ô¼°ÆäÇø¼ä£¬×ø±ê±ê×¢

set(gca,'XTick',[0:0.2:1],'YTick',[0:0.2:1],'ZTick',[0:0.2:1])

axis([0 1 0 1 0 1])

xlabel('$x$','interpreter','latex');ylabel('$y$','interpreter','latex');zlabel('$z$','interpreter','latex','Rotation',360,'position',[-0.1 1 1.1]);

%Í¼ÏóÍø¸ñ£¬µ×Í¼¼Ó°×

grid on

hold on

set(0,'defaultfigurecolor','w')

%Í¼Àý¼°±êÌâ

legend({'{\it\fontname{Bodoni MT} \alpha}=0.5','{\it\fontname{Bodoni MT} \alpha}=0.7','{\it\fontname{Bodoni MT} \alpha}=0.8'},'location','northeast');

title(' Fig.9. The influence of recycling revenue sharing ratio \alpha.','FontWeight','bold','position',[0 0 -0.2]);

% the small figure

axes('position',[0.13 0.32 0.2 0.2]); %Ð¡Í¼µÄ¹Ø¼üÓï¾äÈ·¶¨Ð¡Í¼µÄ´óÐ¡Î»ÖÃ

%¼ÓÉÏ±ê×¢ºÍ¼ýÍ·

text(0.5 ,0.5 ,0.5,'$ESS$','interpreter','latex');

text(0.56 ,0.29 ,0.3,'ESS');

annotation('arrow',[0.55 0.35],[0.35 0.32]);

annotation('arrow',[0.58 0.58],[0.38 0.45]);

% % y-xÐ¡Í¼ÖÐµÄÏßÌõ

%Ð¡Í¼ÖÐµÄÏßÌõ

a=0.5,b=0.8,K=100,R=150,P=20,T=80,F1=150,F2=350,S1=20,S2=15,C1=25,C2=10,C3=55,C4=5;

[t,y]=ode45(@(t,y) manufacturer1(t,y,a,b,K,R,P,T,F1,F2,S1,S2,C1,C2,C3,C4),[0 50],[0.2 0.2 0.2]);

plot3(y(:,1),y(:,2),y(:,3),'r+','linewidth',1);

hold on

a=0.7,b=0.8,K=100,R=150,P=20,T=80,F1=150,F2=350,S1=20,S2=15,C1=25,C2=10,C3=55,C4=5;

[t,y]=ode45(@(t,y) manufacturer1(t,y,a,b,K,R,P,T,F1,F2,S1,S2,C1,C2,C3,C4),[0 50],[0.2 0.2 0.2]);

plot3(y(:,1),y(:,2),y(:,3),'b-','linewidth',1);

hold on

a=0.8,b=0.8,K=100,R=150,P=20,T=80,F1=150,F2=350,S1=20,S2=15,C1=25,C2=10,C3=55,C4=5;

[t,y]=ode45(@(t,y) manufacturer1(t,y,a,b,K,R,P,T,F1,F2,S1,S2,C1,C2,C3,C4),[0 50],[0.2 0.2 0.2]);

plot3(y(:,1),y(:,2),y(:,3),'g--','linewidth',1);

hold on

%×ø±ê¿Ì¶È¼ä¸ô¼°Çø¼ä£¬µ«²»ÏÔÊ¾

set(gca,'XTick',[0:0.2:1],'YTick',[0:0.2:1],'ZTick',[0:0.2:1])

axis([0 1 0 1 0 1])

set(gca,'XTickLabel','','YTickLabel','','ZTickLabel','')

%±³¾°Íø¸ñ²¢ÉèÖÃ°×µ×

grid on

hold on

set(0,'defaultfigurecolor','w')

%Í¼ÏóÏÔÊ¾ÊÓ½Ç£¬ÒÔ¼°ÏàÓ¦µÄ×ø±ê±ê×¢

view([0 90]); %y-xÐ¡Í¼

xlabel('x','position',[0.8 1 0.3])

ylabel('y','position',[0.1 1 0.8],'Rotation',360)

1. **Fig 10. The influence of revenue-sharing coefficients** $\boldsymbol{\beta}$

clc,clear;

figure(10);

%b=0.5

a=0.7,b=0.5,K=100,R=150,P=20,T=80,F1=150,F2=350,S1=20,S2=15,C1=25,C2=10,C3=55,C4=5;

[t,y]=ode45(@(t,y) manufacturer1(t,y,a,b,K,R,P,T,F1,F2,S1,S2,C1,C2,C3,C4),[0 50],[0.2 0.2 0.2]);

plot3(y(:,1),y(:,2),y(:,3),'r+','linewidth',1);

hold on ;

%b=0.7

a=0.7,b=0.7,K=100,R=150,P=20,T=80,F1=150,F2=350,S1=20,S2=15,C1=25,C2=10,C3=55,C4=5;

[t,y]=ode45(@(t,y) manufacturer1(t,y,a,b,K,R,P,T,F1,F2,S1,S2,C1,C2,C3,C4),[0 50],[0.2 0.2 0.2]);

plot3(y(:,1),y(:,2),y(:,3),'b-','linewidth',1);

hold on ;

%b=0.9

a=0.7,b=0.9,K=100,R=150,P=20,T=80,F1=150,F2=350,S1=20,S2=15,C1=25,C2=10,C3=55,C4=5;

[t,y]=ode45(@(t,y) manufacturer1(t,y,a,b,K,R,P,T,F1,F2,S1,S2,C1,C2,C3,C4),[0 50],[0.2 0.2 0.2]);

plot3(y(:,1),y(:,2),y(:,3),'g--','linewidth',1);

hold on ;

%×ø±ê¿Ì¶È¼ä¸ô¼°ÆäÇø¼ä£¬×ø±ê±ê×¢

set(gca,'XTick',[0:0.2:1],'YTick',[0:0.2:1],'ZTick',[0:0.2:1])

axis([0 1 0 1 0 1])

xlabel('$x$','interpreter','latex');ylabel('$y$','interpreter','latex');zlabel('$z$','interpreter','latex','Rotation',360,'position',[-0.1 1 1.1]);

%Í¼ÏóÍø¸ñ£¬µ×Í¼¼Ó°×

grid on

hold on

set(0,'defaultfigurecolor','w')

%Í¼Àý¼°±êÌâ

legend({'{\it\fontname{Bodoni MT} \beta}=0.5','{\it\fontname{Bodoni MT} \beta}=0.7','{\it\fontname{Bodoni MT} \beta}=0.8'},'location','northeast');

title(' Fig.10. The influence of revenue sharing ratio \beta from new products sale','FontWeight','bold','position',[0 0 -0.2]);

% the small figure

axes('position',[0.13 0.32 0.2 0.2]); %Ð¡Í¼µÄ¹Ø¼üÓï¾äÈ·¶¨Ð¡Í¼µÄ´óÐ¡Î»ÖÃ

%¼ÓÉÏ±ê×¢ºÍ¼ýÍ·

text(0.5 ,0.5 ,0.5,'$ESS$','interpreter','latex');

text(0.56 ,0.29 ,0.3,'ESS');

annotation('arrow',[0.55 0.35],[0.35 0.32]);

annotation('arrow',[0.58 0.58],[0.38 0.45]);

% % y-xÐ¡Í¼ÖÐµÄÏßÌõ

%Ð¡Í¼ÖÐµÄÏßÌõ

a=0.7,b=0.5,K=100,R=150,P=20,T=80,F1=150,F2=350,S1=20,S2=15,C1=25,C2=10,C3=55,C4=5;

[t,y]=ode45(@(t,y) manufacturer1(t,y,a,b,K,R,P,T,F1,F2,S1,S2,C1,C2,C3,C4),[0 50],[0.2 0.2 0.2]);

plot3(y(:,1),y(:,2),y(:,3),'r+','linewidth',1);

hold on

a=0.7,b=0.7,K=100,R=150,P=20,T=80,F1=150,F2=350,S1=20,S2=15,C1=25,C2=10,C3=55,C4=5;

[t,y]=ode45(@(t,y) manufacturer1(t,y,a,b,K,R,P,T,F1,F2,S1,S2,C1,C2,C3,C4),[0 50],[0.2 0.2 0.2]);

plot3(y(:,1),y(:,2),y(:,3),'b-','linewidth',1);

hold on

a=0.7,b=0.9,K=100,R=150,P=20,T=80,F1=150,F2=350,S1=20,S2=15,C1=25,C2=10,C3=55,C4=5;

[t,y]=ode45(@(t,y) manufacturer1(t,y,a,b,K,R,P,T,F1,F2,S1,S2,C1,C2,C3,C4),[0 50],[0.2 0.2 0.2]);

plot3(y(:,1),y(:,2),y(:,3),'g--','linewidth',1);

hold on

%×ø±ê¿Ì¶È¼ä¸ô¼°Çø¼ä£¬µ«²»ÏÔÊ¾

set(gca,'XTick',[0:0.2:1],'YTick',[0:0.2:1],'ZTick',[0:0.2:1])

axis([0 1 0 1 0 1])

set(gca,'XTickLabel','','YTickLabel','','ZTickLabel','')

%±³¾°Íø¸ñ²¢ÉèÖÃ°×µ×

grid on

hold on

set(0,'defaultfigurecolor','w')

%Í¼ÏóÏÔÊ¾ÊÓ½Ç£¬ÒÔ¼°ÏàÓ¦µÄ×ø±ê±ê×¢

view([0 90]); %y-xÐ¡Í¼

xlabel('x','position',[0.8 1 0.3])

ylabel('y','position',[0.1 1 0.8],'Rotation',360)

1. **Fig 11. The influence of innovation input cost** $\boldsymbol{T}$ **for manufacturers**

clc,clear;

figure(11);

%,T=50

a=0.7,b=0.8,K=100,R=150,P=20,T=50,F1=150,F2=350,S1=20,S2=15,C1=25,C2=10,C3=55,C4=5;

[t,y]=ode45(@(t,y) manufacturer1(t,y,a,b,K,R,P,T,F1,F2,S1,S2,C1,C2,C3,C4),[0 50],[0.2 0.2 0.2]);

plot3(y(:,1),y(:,2),y(:,3),'r+','linewidth',1);

hold on ;

%,T=80

a=0.7,b=0.8,K=100,R=150,P=20,T=80,F1=150,F2=350,S1=20,S2=15,C1=25,C2=10,C3=55,C4=5;

[t,y]=ode45(@(t,y) manufacturer1(t,y,a,b,K,R,P,T,F1,F2,S1,S2,C1,C2,C3,C4),[0 50],[0.2 0.2 0.2]);

plot3(y(:,1),y(:,2),y(:,3),'b-','linewidth',1);

hold on ;

%,T=100

a=0.7,b=0.8,K=100,R=150,P=20,T=100,F1=150,F2=350,S1=20,S2=15,C1=25,C2=10,C3=55,C4=5;

[t,y]=ode45(@(t,y) manufacturer1(t,y,a,b,K,R,P,T,F1,F2,S1,S2,C1,C2,C3,C4),[0 50],[0.2 0.2 0.2]);

plot3(y(:,1),y(:,2),y(:,3),'g--','linewidth',1);

hold on ;

%×ø±ê¿Ì¶È¼ä¸ô¼°ÆäÇø¼ä£¬×ø±ê±ê×¢

set(gca,'XTick',[0:0.2:1],'YTick',[0:0.2:1],'ZTick',[0:0.2:1])

axis([0 1 0 1 0 1])

xlabel('$x$','interpreter','latex');ylabel('$y$','interpreter','latex');zlabel('$z$','interpreter','latex','Rotation',360,'position',[-0.1 1 1.1]);

%Í¼ÏóÍø¸ñ£¬µ×Í¼¼Ó°×

grid on

hold on

set(0,'defaultfigurecolor','w')

%Í¼Àý¼°±êÌâ

legend({'{\it\fontname{Bodoni MT} T}=50','{\it\fontname{Bodoni MT} T}=80','{\it\fontname{Bodoni MT} T}=100'},'location','northeast');

title(' Fig.11. The influence of innovation input cost T for manufacturers','FontWeight','bold','position',[0 0 -0.2]);

% the small figure

axes('position',[0.13 0.32 0.2 0.2]); %Ð¡Í¼µÄ¹Ø¼üÓï¾äÈ·¶¨Ð¡Í¼µÄ´óÐ¡Î»ÖÃ

%¼ÓÉÏ±ê×¢ºÍ¼ýÍ·

text(0.5 ,0.5 ,0.5,'$ESS$','interpreter','latex');

text(0.56 ,0.29 ,0.3,'ESS');

annotation('arrow',[0.55 0.35],[0.35 0.32]);

annotation('arrow',[0.58 0.58],[0.38 0.45]);

% % y-xÐ¡Í¼ÖÐµÄÏßÌõ

%Ð¡Í¼ÖÐµÄÏßÌõ

a=0.7,b=0.8,K=100,R=150,P=20,T=50,F1=150,F2=350,S1=20,S2=15,C1=25,C2=10,C3=55,C4=5;

[t,y]=ode45(@(t,y) manufacturer1(t,y,a,b,K,R,P,T,F1,F2,S1,S2,C1,C2,C3,C4),[0 50],[0.2 0.2 0.2]);

plot3(y(:,1),y(:,2),y(:,3),'r+','linewidth',1);

hold on

a=0.7,b=0.8,K=100,R=150,P=20,T=80,F1=150,F2=350,S1=20,S2=15,C1=25,C2=10,C3=55,C4=5;

[t,y]=ode45(@(t,y) manufacturer1(t,y,a,b,K,R,P,T,F1,F2,S1,S2,C1,C2,C3,C4),[0 50],[0.2 0.2 0.2]);

plot3(y(:,1),y(:,2),y(:,3),'b-','linewidth',1);

hold on

a=0.7,b=0.8,K=100,R=150,P=20,T=100,F1=150,F2=350,S1=20,S2=15,C1=25,C2=10,C3=55,C4=5;

[t,y]=ode45(@(t,y) manufacturer1(t,y,a,b,K,R,P,T,F1,F2,S1,S2,C1,C2,C3,C4),[0 50],[0.2 0.2 0.2]);

plot3(y(:,1),y(:,2),y(:,3),'g--','linewidth',1);

hold on

%×ø±ê¿Ì¶È¼ä¸ô¼°Çø¼ä£¬µ«²»ÏÔÊ¾

set(gca,'XTick',[0:0.2:1],'YTick',[0:0.2:1],'ZTick',[0:0.2:1])

axis([0 1 0 1 0 1])

set(gca,'XTickLabel','','YTickLabel','','ZTickLabel','')

%±³¾°Íø¸ñ²¢ÉèÖÃ°×µ×

grid on

hold on

set(0,'defaultfigurecolor','w')

%Í¼ÏóÏÔÊ¾ÊÓ½Ç£¬ÒÔ¼°ÏàÓ¦µÄ×ø±ê±ê×¢

view([0 90]); %y-xÐ¡Í¼

xlabel('x','position',[0.8 1 0.3])

ylabel('y','position',[0.1 1 0.8],'Rotation',360)%% Í¼13 ¹Ë¿ÍÔÚÒÔ¾É»»ÐÂÖÐµÄ¼ÛÖµÔöÖµK µÄÓ°Ïì£¨Í¼4µÄÐ¡Í¼µÄ±ê×¢ÊÇ¿ÉÒÔËæÒâÒÆ¶¯µÄ£¬ÆäËûÍ¼²»ÐÐ¡£ÁíÍâ£¬Í¼6µÄÎÄ±¾º¯ÊýÎÞ·¨ÏÔÊ¾£¬ÆäËûÍ¼¿ÉÒÔ¡££©

1. **Fig 12. The influence of subsidie** $\boldsymbol{S}_{\mathbf{1}}$**from manufacturers**

clc,clear;

figure(12);

%,S1=10

a=0.7,b=0.8,K=100,R=150,P=20,T=80,F1=150,F2=350,S1=10,S2=15,C1=25,C2=10,C3=55,C4=5;

[t,y]=ode45(@(t,y) manufacturer1(t,y,a,b,K,R,P,T,F1,F2,S1,S2,C1,C2,C3,C4),[0 50],[0.2 0.2 0.2]);

plot3(y(:,1),y(:,2),y(:,3),'r+','linewidth',1);

hold on ;

%,S1=20

a=0.7,b=0.8,K=100,R=150,P=20,T=80,F1=150,F2=350,S1=20,S2=15,C1=25,C2=10,C3=55,C4=5;

[t,y]=ode45(@(t,y) manufacturer1(t,y,a,b,K,R,P,T,F1,F2,S1,S2,C1,C2,C3,C4),[0 50],[0.2 0.2 0.2]);

plot3(y(:,1),y(:,2),y(:,3),'b-','linewidth',1);

hold on ;

%,S1=30

a=0.7,b=0.8,K=100,R=150,P=20,T=80,F1=150,F2=350,S1=30,S2=15,C1=25,C2=10,C3=55,C4=5;

[t,y]=ode45(@(t,y) manufacturer1(t,y,a,b,K,R,P,T,F1,F2,S1,S2,C1,C2,C3,C4),[0 50],[0.2 0.2 0.2]);

plot3(y(:,1),y(:,2),y(:,3),'g--','linewidth',1);

hold on ;

%×ø±ê¿Ì¶È¼ä¸ô¼°ÆäÇø¼ä£¬×ø±ê±ê×¢

set(gca,'XTick',[0:0.2:1],'YTick',[0:0.2:1],'ZTick',[0:0.2:1])

axis([0 1 0 1 0 1])

xlabel('$x$','interpreter','latex');ylabel('$y$','interpreter','latex');zlabel('$z$','interpreter','latex','Rotation',360,'position',[-0.1 1 1.1]);

%Í¼ÏóÍø¸ñ£¬µ×Í¼¼Ó°×

grid on

hold on

set(0,'defaultfigurecolor','w')

%Í¼Àý¼°±êÌâ

legend({'{\it\fontname{Bodoni MT}S_{1}}=10','{\it\fontname{Bodoni MT}S_{1}}=20','{\it\fontname{Bodoni MT}S_{1}}=30'},'location','northeast');

title(' Fig.12. The influence of subsidie S_{1} of recyclers from manufacturers','FontWeight','bold','position',[0 0 -0.2]);

% the small figure

axes('position',[0.13 0.32 0.2 0.2]); %Ð¡Í¼µÄ¹Ø¼üÓï¾äÈ·¶¨Ð¡Í¼µÄ´óÐ¡Î»ÖÃ

%¼ÓÉÏ±ê×¢ºÍ¼ýÍ·

text(0.5 ,0.5 ,0.5,'$ESS$','interpreter','latex');

text(0.56 ,0.29 ,0.3,'ESS');

annotation('arrow',[0.55 0.35],[0.35 0.32]);

annotation('arrow',[0.58 0.58],[0.38 0.45]);

% % y-xÐ¡Í¼ÖÐµÄÏßÌõ

%Ð¡Í¼ÖÐµÄÏßÌõ

a=0.7,b=0.8,K=100,R=150,P=20,T=80,F1=150,F2=350,S1=10,S2=15,C1=25,C2=10,C3=55,C4=5;

[t,y]=ode45(@(t,y) manufacturer1(t,y,a,b,K,R,P,T,F1,F2,S1,S2,C1,C2,C3,C4),[0 50],[0.2 0.2 0.2]);

plot3(y(:,1),y(:,2),y(:,3),'r+','linewidth',1);

hold on

a=0.7,b=0.8,K=100,R=150,P=20,T=80,F1=150,F2=350,S1=20,S2=15,C1=25,C2=10,C3=55,C4=5;

[t,y]=ode45(@(t,y) manufacturer1(t,y,a,b,K,R,P,T,F1,F2,S1,S2,C1,C2,C3,C4),[0 50],[0.2 0.2 0.2]);

plot3(y(:,1),y(:,2),y(:,3),'b-','linewidth',1);

hold on

a=0.7,b=0.8,K=100,R=150,P=20,T=80,F1=150,F2=350,S1=30,S2=15,C1=25,C2=10,C3=55,C4=5;

[t,y]=ode45(@(t,y) manufacturer1(t,y,a,b,K,R,P,T,F1,F2,S1,S2,C1,C2,C3,C4),[0 50],[0.2 0.2 0.2]);

plot3(y(:,1),y(:,2),y(:,3),'g--','linewidth',1);

hold on

%×ø±ê¿Ì¶È¼ä¸ô¼°Çø¼ä£¬µ«²»ÏÔÊ¾

set(gca,'XTick',[0:0.2:1],'YTick',[0:0.2:1],'ZTick',[0:0.2:1])

axis([0 1 0 1 0 1])

set(gca,'XTickLabel','','YTickLabel','','ZTickLabel','')

%±³¾°Íø¸ñ²¢ÉèÖÃ°×µ×

grid on

hold on

set(0,'defaultfigurecolor','w')

%Í¼ÏóÏÔÊ¾ÊÓ½Ç£¬ÒÔ¼°ÏàÓ¦µÄ×ø±ê±ê×¢

view([0 90]); %y-xÐ¡Í¼

xlabel('x','position',[0.8 1 0.3])

ylabel('y','position',[0.1 1 0.8],'Rotation',360)

1. **Fig 13. The influence of subsidie** $\boldsymbol{S}_{\mathbf{2}}$**from manufacturers**

subplot(2,2,2);

%,S2=10

a=0.7,b=0.8,K=100,R=150,P=20,T=80,F1=150,F2=350,S1=20,S2=10,C1=25,C2=10,C3=55,C4=5;

[t,y]=ode45(@(t,y) manufacturer1(t,y,a,b,K,R,P,T,F1,F2,S1,S2,C1,C2,C3,C4),[0 50],[0.2 0.2 0.2]);

plot3(y(:,1),y(:,2),y(:,3),'r+','linewidth',1);

hold on ;

%,S2=20

a=0.7,b=0.8,K=100,R=150,P=20,T=80,F1=150,F2=350,S1=20,S2=20,C1=25,C2=10,C3=55,C4=5;

[t,y]=ode45(@(t,y) manufacturer1(t,y,a,b,K,R,P,T,F1,F2,S1,S2,C1,C2,C3,C4),[0 50],[0.2 0.2 0.2]);

plot3(y(:,1),y(:,2),y(:,3),'b-','linewidth',1);

hold on ;

%,S2=30

a=0.7,b=0.8,K=100,R=150,P=20,T=80,F1=150,F2=350,S1=20;S2=30,C1=25,C2=10,C3=55,C4=5;

[t,y]=ode45(@(t,y) manufacturer1(t,y,a,b,K,R,P,T,F1,F2,S1,S2,C1,C2,C3,C4),[0 50],[0.2 0.2 0.2]);

plot3(y(:,1),y(:,2),y(:,3),'g--','linewidth',1);

hold on ;

%×ø±ê¿Ì¶È¼ä¸ô¼°ÆäÇø¼ä£¬×ø±ê±ê×¢

set(gca,'XTick',[0:0.2:1],'YTick',[0:0.2:1],'ZTick',[0:0.2:1])

axis([0 1 0 1 0 1])

xlabel('$x$','interpreter','latex');ylabel('$y$','interpreter','latex');zlabel('$z$','interpreter','latex','Rotation',360,'position',[-0.1 1 1.1]);

%Í¼ÏóÍø¸ñ£¬µ×Í¼¼Ó°×

grid on

hold on

set(0,'defaultfigurecolor','w')

%Í¼Àý¼°±êÌâ

legend({'{\it\fontname{Bodoni MT}S_{2}}=10','{\it\fontname{Bodoni MT}S_{2}}=20','{\it\fontname{Bodoni MT}S_{2}}=30'},'location','northeast');

title(' Fig.13. The influence of subsidie S_{2} of customers from manufacturers','FontWeight','bold','position',[0 0 -0.2]);

% the small figure

axes('position',[0.13 0.32 0.2 0.2]); %Ð¡Í¼µÄ¹Ø¼üÓï¾äÈ·¶¨Ð¡Í¼µÄ´óÐ¡Î»ÖÃ

%¼ÓÉÏ±ê×¢ºÍ¼ýÍ·

text(0.5 ,0.5 ,0.5,'$ESS$','interpreter','latex');

text(0.56 ,0.29 ,0.3,'ESS');

annotation('arrow',[0.55 0.35],[0.35 0.32]);

annotation('arrow',[0.58 0.58],[0.38 0.45]);

% % y-xÐ¡Í¼ÖÐµÄÏßÌõ

%Ð¡Í¼ÖÐµÄÏßÌõ

a=0.7,b=0.8,K=100,R=150,P=20,T=80,F1=150,F2=350,S1=20,S2=10,C1=25,C2=10,C3=55,C4=5;

[t,y]=ode45(@(t,y) manufacturer1(t,y,a,b,K,R,P,T,F1,F2,S1,S2,C1,C2,C3,C4),[0 50],[0.2 0.2 0.2]);

plot3(y(:,1),y(:,2),y(:,3),'r+','linewidth',1);

hold on

a=0.7,b=0.8,K=100,R=150,P=20,T=80,F1=150,F2=350,S1=20,S2=20,C1=25,C2=10,C3=55,C4=5;

[t,y]=ode45(@(t,y) manufacturer1(t,y,a,b,K,R,P,T,F1,F2,S1,S2,C1,C2,C3,C4),[0 50],[0.2 0.2 0.2]);

plot3(y(:,1),y(:,2),y(:,3),'b-','linewidth',1);

hold on

a=0.7,b=0.8,K=100,R=150,P=20,T=80,F1=150,F2=350,S1=20,S2=30,C1=25,C2=10,C3=55,C4=5;

[t,y]=ode45(@(t,y) manufacturer1(t,y,a,b,K,R,P,T,F1,F2,S1,S2,C1,C2,C3,C4),[0 50],[0.2 0.2 0.2]);

plot3(y(:,1),y(:,2),y(:,3),'g--','linewidth',1);

hold on

%×ø±ê¿Ì¶È¼ä¸ô¼°Çø¼ä£¬µ«²»ÏÔÊ¾

set(gca,'XTick',[0:0.2:1],'YTick',[0:0.2:1],'ZTick',[0:0.2:1])

axis([0 1 0 1 0 1])

set(gca,'XTickLabel','','YTickLabel','','ZTickLabel','')

%±³¾°Íø¸ñ²¢ÉèÖÃ°×µ×

grid on

hold on

set(0,'defaultfigurecolor','w')

%Í¼ÏóÏÔÊ¾ÊÓ½Ç£¬ÒÔ¼°ÏàÓ¦µÄ×ø±ê±ê×¢

view([0 90]); %y-xÐ¡Í¼

xlabel('x','position',[0.8 1 0.3])

ylabel('y','position',[0.1 1 0.8],'Rotation',360)

1. **Fig 14. The influence of benefit** $\boldsymbol{\psi}_{\boldsymbol{1}}$ **for manufacturers after adopting GI**

clc,clear;

figure(14);

%,F1=150

a=0.7,b=0.8,K=100,R=150,P=20,T=80,F1=150,F2=350,S1=20,S2=15,C1=25,C2=10,C3=55,C4=5;

[t,y]=ode45(@(t,y) manufacturer1(t,y,a,b,K,R,P,T,F1,F2,S1,S2,C1,C2,C3,C4),[0 50],[0.2 0.2 0.2]);

plot3(y(:,1),y(:,2),y(:,3),'r+','linewidth',1);

hold on ;

%,S2=20

a=0.7,b=0.8,K=100,R=150,P=20,T=80,F1=200,F2=350,S1=20,S2=15,C1=25,C2=10,C3=55,C4=5;

[t,y]=ode45(@(t,y) manufacturer1(t,y,a,b,K,R,P,T,F1,F2,S1,S2,C1,C2,C3,C4),[0 50],[0.2 0.2 0.2]);

plot3(y(:,1),y(:,2),y(:,3),'b-','linewidth',1);

hold on ;

%,S2=30

a=0.7,b=0.8,K=100,R=150,P=20,T=80,F1=300,F2=350,S1=20,S2=15,C1=25,C2=10,C3=55,C4=5;

[t,y]=ode45(@(t,y) manufacturer1(t,y,a,b,K,R,P,T,F1,F2,S1,S2,C1,C2,C3,C4),[0 50],[0.2 0.2 0.2]);

plot3(y(:,1),y(:,2),y(:,3),'g--','linewidth',1);

hold on ;

%×ø±ê¿Ì¶È¼ä¸ô¼°ÆäÇø¼ä£¬×ø±ê±ê×¢

set(gca,'XTick',[0:0.2:1],'YTick',[0:0.2:1],'ZTick',[0:0.2:1])

axis([0 1 0 1 0 1])

xlabel('$x$','interpreter','latex');ylabel('$y$','interpreter','latex');zlabel('$z$','interpreter','latex','Rotation',360,'position',[-0.1 1 1.1]);

%Í¼ÏóÍø¸ñ£¬µ×Í¼¼Ó°×

grid on

hold on

set(0,'defaultfigurecolor','w')

%Í¼Àý¼°±êÌâ

legend({'{\it\fontname{Bodoni MT}\psi_{1}}=150','{\it\fontname{Bodoni MT_}\psi_{1}}=200','{\it\fontname{Bodoni MT}\psi_{1}}=300'},'location','northeast');

title(' Fig.14. The influence of social and brand benefit \psi_{1} from GI','FontWeight','bold','position',[0 0 -0.2]);

% the small figure

axes('position',[0.13 0.32 0.2 0.2]); %Ð¡Í¼µÄ¹Ø¼üÓï¾äÈ·¶¨Ð¡Í¼µÄ´óÐ¡Î»ÖÃ

%¼ÓÉÏ±ê×¢ºÍ¼ýÍ·

text(0.5 ,0.5 ,0.5,'$ESS$','interpreter','latex');

text(0.56 ,0.29 ,0.3,'ESS');

annotation('arrow',[0.55 0.35],[0.35 0.32]);

annotation('arrow',[0.58 0.58],[0.38 0.45]);

% % y-xÐ¡Í¼ÖÐµÄÏßÌõ

%Ð¡Í¼ÖÐµÄÏßÌõ

a=0.7,b=0.8,K=100,R=150,P=20,T=80,F1=150,F2=350,S1=20,S2=15,C1=25,C2=10,C3=55,C4=5;

[t,y]=ode45(@(t,y) manufacturer1(t,y,a,b,K,R,P,T,F1,F2,S1,S2,C1,C2,C3,C4),[0 50],[0.2 0.2 0.2]);

plot3(y(:,1),y(:,2),y(:,3),'r+','linewidth',1);

hold on

a=0.7,b=0.8,K=100,R=150,P=20,T=80,F1=200,F2=350,S1=20,S2=15,C1=25,C2=10,C3=55,C4=5;

[t,y]=ode45(@(t,y) manufacturer1(t,y,a,b,K,R,P,T,F1,F2,S1,S2,C1,C2,C3,C4),[0 50],[0.2 0.2 0.2]);

plot3(y(:,1),y(:,2),y(:,3),'b-','linewidth',1);

hold on

a=0.7,b=0.8,K=100,R=150,P=20,T=80,F1=300,F2=350,S1=20,S2=15,C1=25,C2=10,C3=55,C4=5;

[t,y]=ode45(@(t,y) manufacturer1(t,y,a,b,K,R,P,T,F1,F2,S1,S2,C1,C2,C3,C4),[0 50],[0.2 0.2 0.2]);

plot3(y(:,1),y(:,2),y(:,3),'g--','linewidth',1);

hold on

%×ø±ê¿Ì¶È¼ä¸ô¼°Çø¼ä£¬µ«²»ÏÔÊ¾

set(gca,'XTick',[0:0.2:1],'YTick',[0:0.2:1],'ZTick',[0:0.2:1])

axis([0 1 0 1 0 1])

set(gca,'XTickLabel','','YTickLabel','','ZTickLabel','')

%±³¾°Íø¸ñ²¢ÉèÖÃ°×µ×

grid on

hold on

set(0,'defaultfigurecolor','w')

%Í¼ÏóÏÔÊ¾ÊÓ½Ç£¬ÒÔ¼°ÏàÓ¦µÄ×ø±ê±ê×¢

view([0 90]); %y-xÐ¡Í¼

xlabel('x','position',[0.8 1 0.3])

ylabel('y','position',[0.1 1 0.8],'Rotation',360)

1. **Fig 15. The result of 50 evolutions of array 1**

clc,clear;

a=0.7,b=0.8,K=100,R=150,P=20,T=80,F1=150,F2=350,S1=20,S2=15,C1=25,C2=10,C3=55,C4=5;

y1=K-P+S2-C4

y2=F1-C1-S1-S2-T+C2

Y3=(1-b)* F2+(1-a)*R+S1-C3,

%% Í¼16ÕûÌåÑÝ»¯£¨Í¼6µÄÐ¡Í¼µÄ±ê×¢ÊÇ¿ÉÒÔËæÒâÒÆ¶¯µÄ£¬ÆäËûÍ¼²»ÐÐ¡£ÁíÍâ£¬Í¼6µÄÎÄ±¾º¯ÊýÎÞ·¨ÏÔÊ¾£¬ÆäËûÍ¼¿ÉÒÔ¡££©

%% ÈýÎ¬x-y-zÊý×é

%²»Í¬³õÊ¼²ßÂÔ×éºÏÑÝ»¯Í¼

%%

%Í¼11£¬Êý×é1

clc,clear;

figure(11);

a=0.7,b=0.8,K=100,R=150,P=20,T=80,F1=150,F2=350,S1=20,S2=15,C1=25,C2=10,C3=55,C4=5;

for i=0.1:0.2:1

for j=0.1:0.2:1

for k=0.1:0.2:1

[t,y]=ode45(@(t,y) manufacturer1(t,y,a,b,K,R,P,T,F1,F2,S1,S2,C1,C2,C3,C4),[0 50],[i j k]);

%plot3(y(:,1),y(:,2),y(:,3),'linewidth',1);

plot3(y(:,1),y(:,2),y(:,3),'rp','linewidth',1); %°ÑÑÕÉ«¸ÄÎªºìÉ«£¬ÏßÐÍ¸ÄÎªÎå½ÇÐÇ¡£

set(gca,'XTick',[0:0.2:1],'YTick',[0:0.2:1],'ZTick',[0:0.2:1])

hold on

axis([0 1 0 1 0 1])

view([45 10])

end

end

end

grid on

hold on

xlabel('x','Rotation',0);

ylabel('y','Rotation',0);

zlabel('z','Rotation',360,'position',[0 0 1.05]);

title('Í¼ 15 Êý×é1ÑÝ»¯50´Î½á¹û','FontWeight','bold','position',[1 0 -0.13]);

%%

%Í¼12£¬Êý×é2

clc,clear;

figure(12);

a=0.7,b=0.8,K=100,R=150,P=20,T=80,F1=150,F2=350,S1=20,S2=15,C1=25,C2=10,C3=55,C4=5;

for i=0.1:0.2:1

for j=0.1:0.2:1

for k=0.1:0.2:1

[t,y]=ode45(@(t,y) manufacturer1(t,y,a,b,K,R,P,T,F1,F2,S1,S2,C1,C2,C3,C4),[0 50],[i j k]);

plot3(y(:,1),y(:,2),y(:,3),'linewidth',1);

set(gca,'XTick',[0:0.2:1],'YTick',[0:0.2:1],'ZTick',[0:0.2:1])

hold on

axis([0 1 0 1 0 1])

view([45 10])

end

end

end

grid on

hold on

xlabel('x','Rotation',0);

ylabel('y','Rotation',0);

zlabel('z','Rotation',360,'position',[0 0 1.05]);

%title('Fig.16. The result of 50 evolutions of array 1','FontWeight','bold','position',[1 0 -0.13]);
