# Supplementary material for: Behavioral choice of manufacturers, recyclers and customers in Trade-In Programs
Source: PLoS One. 2024 Dec 30;19(12):e0316344. doi: 10.1371/journal.pone.0316344 (PMC11684666; doi:10.1371/journal.pone.0316344)
Supplement: S1 File — (ZIP) [file pone.0316344.s001.zip › Programs/Replication dynamic equations.docx]

%%%%%%% **Replication dynamic equations**

function dydt=manufacturer1(t,y,a,b,K,R,P,T,F1,F2,S1,S2,C1,C2,C3,C4) %º¯Êý¶¨ÒåÇ°ºó±£³ÖÒ»ÖÂ£¬y¸ÄÎªx£¬ÄÇÃ´ºóÃæy(1)¸ÄÎªx(1)£¬ÒÔ´ËÀàÍÆ¡£

dydt=zeros(3,1);%Ë«·½Ôò¸ÄÎª£¨2,1£©£¬ËÄ·½Ôò¸ÄÎª£¨4,1£©

%²ÎÊý×ÖÄ¸Èç¹ûÊÇÏ£À°×ÖÄ¸£¬¾¡Á¿ÓÃÆä¶ÁÒôÆ´Ð´£¬¿ÉÒÔ°Ù¶È²é¿´

dydt(1)=y(1)*(y(1)-1)*(C1-C2-F1+T+S1*y(2)+S2*y(3));

dydt(2)=y(2)*(y(2)-1)*(C3-F2*y(3)-S1*y(1)-R*y(3)+F2*b*y(3)+R*a*y(3));

dydt(3)=y(3)*(y(3)-1)*(C4-K+P-S2*y(1));

end
